# Supplementary material for: A comparative study of blood cell count in four automated hematology analyzers: An evaluation of the impact of preanalytical factors
Source: PLoS One. 2024 May 24;19(5):e0301845. doi: 10.1371/journal.pone.0301845 (PMC11125483; doi:10.1371/journal.pone.0301845)
Supplement: S1 Table — (PDF) [file pone.0301845.s001.pdf]

Donor #	Sex	Age (yrs)	Disease status	Medication for asthma
1	M	NR	Healthy	NA
2	F	NR	Healthy	NA
3	F	NR	Healthy atopic	NA
4	F	NR	Healthy	NA
5	F	NR	Healthy atopic	NA
6	M	NR	Healthy atopic	NA
7	M	NR	Healthy atopic	NA
8	M	NR	Healthy	NA
9	M	NR	Healthy	NA
10	F	NR	Healthy	NA
11	F	NR	Healthy atopic	NA
12	F	NR	Healthy atopic	NA
13	M	52	Eosinophilic asthma	ICS, SABA
14	F	65	Eosinophilic asthma	ICS/LABA, SABA
15	F	70	Eosinophilic asthma	NA
16	F	43	Eosinophilic asthma	NA
17	M	59	Eosinophilic asthma	ICS/LABA
18	M	47	Eosinophilic asthma	ICS/LABA, SABA

NA: not applicable; NR: not reported
