# Supplementary material for: A comparative study of blood cell count in four automated hematology analyzers: An evaluation of the impact of preanalytical factors
Source: PLoS One. 2024 May 24;19(5):e0301845. doi: 10.1371/journal.pone.0301845 (PMC11125483; doi:10.1371/journal.pone.0301845)
Supplement: S2 Table — (PDF) [file pone.0301845.s002.pdf]

Marker	Antibody	Supplier	Catalog no
Live/dead	LIVE/DEAD™ Fixable Aqua Dead Cell Stain Kit	ThermoFisher	L34957
Fc	Mouse BD Fc Block	BD Biosciences	553142
CD11b	CD11b-PE	BD Biosciences	555388
CD123	CD123-PeCy7	BD Biosciences	560826
CD62L	CD62L-APC	BD Biosciences	559772
HLA-DR	HLA-DR-BV786	BD Biosciences	564041
CD14	CD14-BV605	BD Biosciences	564054
CD16	CD16-alexa700	BD Biosciences	560713
FcεR1	FcER1-FITC	Biolegend	334608
Sigleg-8	Siglec-8-PECF594	Biolegend	347109
CD66b	CD66b-PerCpCy5.5	Biolegend	305108
Counting beads	CountBright Absolute Counting Beads	ThermoFisher	C36950
**% eos at 3h**	**% eos at 24h**	**% eos at 48h**	**% eos at 72h**
	3.99±2.74 (4°C)	4.31±3.00 (4°C)	5.90±4.86 (4°C)
3.73±3.31 (20°C)	1.88±1.65 (20°C)	1.02±1.05 (20°C)	1.05±0.77 (20°C)
	2.01±1.85 (30°C)	1.25±1.55 (30°C)	0.25±0.20 (30°C)
