# Supplementary material for: A comparative study of blood cell count in four automated hematology analyzers: An evaluation of the impact of preanalytical factors
Source: PLoS One. 2024 May 24;19(5):e0301845. doi: 10.1371/journal.pone.0301845 (PMC11125483; doi:10.1371/journal.pone.0301845)
Supplement: S3 Table — (PDF) [file pone.0301845.s003.pdf]

Instrument	Instrument	Cell type	Difference	95% CI	p-value
Siemens Advia 2120i	Beckman CoulterDxH900	Platelets	9.417	(-0.099, 18.932)	0.052
		Neutrophils	-0.008	(-0.257, 0.241)	0.950
		Lymphocytes	-0.030	(-0.169, 0.110)	0.677
		Eosinophils	-0.003	(-0.064, 0.058)	0.923
		Monocytes	-0.041	(-0.143, 0.062)	0.435
		Basophils	-0.031	(-0.116, 0.053)	0.468
	Abbott CELL-DYN Sapphire	Platelets	-23.018	(-43.440, -2.596)	0.027
		Neutrophils	0.058	(-0.146, 0.261)	0.577
		Lymphocytes	-0.176	(-0.301, -0.050)	0.006
		Eosinophils	-0.031	(-0.113, 0.051)	0.458
		Monocytes	-0.030	(-0.129, 0.070)	0.557
		Basophils	-0.011	(-0.099, 0.077)	0.803
	Sysmex XN-1000V	Platelets	-10.556	(-27.248, 6.137)	0.215
		Neutrophils	-0.003	(-0.275, 0.270)	0.984
		Lymphocytes	-0.167	(-0.312, -0.022)	0.024
		Eosinophils	-0.008	(-0.129, 0.113)	0.893
		Monocytes	-0.063	(-0.192, 0.067)	0.342
		Basophils	-0.008	(-0.094, 0.077)	0.849
Beckman Coulter DxH900	Abbott CELL-DYN Sapphire	Platelets	-32.435	(-52.742, -12.128)	0.002
		Neutrophils	0.066	(-0.168, 0.300)	0.581
		Lymphocytes	-0.146	(-0.319, 0.027)	0.098
		Eosinophils	-0.028	(-0.118, 0.062)	0.540
		Monocytes	0.011	(-0.060, 0.082)	0.762
		Basophils	0.020	(-0.009, 0.050)	0.180
	Sysmex XN-1000V	Platelets	-19.972	(-36.522, -3.422)	0.018
		Neutrophils	0.005	(-0.291, 0.301)	0.972
		Lymphocytes	-0.137	(-0.325, 0.051)	0.153
		Eosinophils	-0.005	(-0.132, 0.121)	0.934

Instrument	Instrument	Cell type	Difference	95% CI	p-value
		Monocytes	-0.022	(-0.131, 0.087)	0.691
		Basophils	0.023	(0.001, 0.045)	0.038
Abbott CELL-DYN Sapphire	Sysmex XN-1000V	Platelets	12.463	(-11.995, 36.920)	0.317
		Neutrophils	-0.061	(-0.319, 0.198)	0.646
		Lymphocytes	0.009	(-0.169, 0.187)	0.921
		Eosinophils	0.023	(-0.115, 0.160)	0.747
		Monocytes	-0.033	(-0.139, 0.073)	0.543
		Basophils	0.003	(-0.030, 0.035)	0.864

Baseline (3h) mean per cell type for comparison was obtained from the Siemens Advia 2120i, Beckman Coulter DxH900 and Sysmex XN-1000V based on 18 donors. Greyed values indicate difference of statistical significance between two platforms
