# Supplementary material for: A comparative study of blood cell count in four automated hematology analyzers: An evaluation of the impact of preanalytical factors
Source: PLoS One. 2024 May 24;19(5):e0301845. doi: 10.1371/journal.pone.0301845 (PMC11125483; doi:10.1371/journal.pone.0301845)
Supplement: S4 Table — (PDF) [file pone.0301845.s004.pdf]

A Platelets

Analyzer	Time	Temperature	Instrument	Difference	95% CI	p-value
Siemens Advia 2120i	6	4	Siemens Advia 2120i	-3.639	(-12.103, 4.826)	0.399
		20	Siemens Advia 2120i	0.722	(-7.742, 9.187)	0.867
		30	Siemens Advia 2120i	5.194	(-3.270, 13.659)	0.229
		37	Siemens Advia 2120i	8.278	(-0.187, 16.742)	0.055
	24	4	Siemens Advia 2120i	-4.583	(-13.048, 3.881)	0.288
		20	Siemens Advia 2120i	-6.306	(-14.770, 2.159)	0.144
		30	Siemens Advia 2120i	0.389	(-8.076, 8.853)	0.928
		37	Siemens Advia 2120i	2.556	(-5.909, 11.020)	0.553
	48	4	Siemens Advia 2120i	3.278	(-5.187, 11.742)	0.447
		20	Siemens Advia 2120i	3.056	(-5.409, 11.520)	0.479
		30	Siemens Advia 2120i	3.917	(-4.548, 12.381)	0.364
		37	Siemens Advia 2120i	-4.000	(-12.465, 4.465)	0.354
	72	4	Siemens Advia 2120i	16.722	(8.258, 25.187)	<.001
		20	Siemens Advia 2120i	4.472	(-3.992, 12.937)	0.300
		30	Siemens Advia 2120i	1.389	(-7.076, 9.853)	0.747
		37	Siemens Advia 2120i	-14.250	(-22.715, -5.785)	0.001
Beckman Coulter DxH900	6	4	Beckman Coulter DxH900	-0.594	(-8.625, 7.436)	0.884
		20	Beckman Coulter DxH900	1.067	(-6.964, 9.097)	0.794
		30	Beckman Coulter DxH900	6.489	(-1.542, 14.520)	0.113
		37	Beckman Coulter DxH900	6.425	(-1.606, 14.456)	0.117
	24	4	Beckman Coulter DxH900	-0.682	(-8.751, 7.388)	0.868
		20	Beckman Coulter DxH900	4.275	(-3.756, 12.306)	0.296
		30	Beckman Coulter DxH900	0.433	(-7.597, 8.464)	0.916
		37	Beckman Coulter DxH900	5.653	(-2.378, 13.683)	0.167
	48	4	Beckman Coulter DxH900	4.036	(-3.995, 12.067)	0.324
		20	Beckman Coulter DxH900	0.289	(-7.742, 8.320)	0.944
		30	Beckman Coulter DxH900	0.100	(-7.931, 8.131)	0.980
		37	Beckman Coulter DxH900	6.883	(-1.147, 14.914)	0.093

Analyzer	Time	Temperature	Instrument	Difference	95% CI	p-value
	72	4	Beckman Coulter DxH900	14.150	(6.119, 22.181)	<.001
		20	Beckman Coulter DxH900	-4.272	(-12.303, 3.758)	0.296
		30	Beckman Coulter DxH900	0.886	(-7.145, 8.917)	0.828
		37	Beckman Coulter DxH900	3.283	(-4.747, 11.314)	0.422
Abbott CELL-DYN Sapphire	6	4	Abbott CELL-DYN Sapphire	-1.917	(-29.121, 25.288)	0.889
		20	Abbott CELL-DYN Sapphire	3.667	(-23.538, 30.871)	0.790
		30	Abbott CELL-DYN Sapphire	17.833	(-9.371, 45.038)	0.197
		37	Abbott CELL-DYN Sapphire	28.083	(0.879, 55.288)	0.043
	24	4	Abbott CELL-DYN Sapphire	3.083	(-24.121, 30.288)	0.823
		20	Abbott CELL-DYN Sapphire	18.250	(-8.954, 45.454)	0.187
		30	Abbott CELL-DYN Sapphire	53.250	(26.046, 80.454)	<.001
		37	Abbott CELL-DYN Sapphire	44.742	(17.537, 71.946)	0.001
	48	4	Abbott CELL-DYN Sapphire	1.018	(-28.371, 30.407)	0.945
		20	Abbott CELL-DYN Sapphire	25.352	(-4.038, 54.741)	0.090
		30	Abbott CELL-DYN Sapphire	54.241	(24.851, 83.630)	<.001
		37	Abbott CELL-DYN Sapphire	33.285	(3.896, 62.674)	0.027
Sysmex XN-1000V	6	4	Sysmex XN-1000V	-0.778	(-19.401, 17.846)	0.935
		20	Sysmex XN-1000V	-2.111	(-20.735, 16.513)	0.824
		30	Sysmex XN-1000V	16.972	(-1.651, 35.596)	0.074
		37	Sysmex XN-1000V	29.694	(11.071, 48.318)	0.002
	24	4	Sysmex XN-1000V	-2.412	(-21.125, 16.300)	0.800
		20	Sysmex XN-1000V	-9.139	(-27.763, 9.485)	0.336
		30	Sysmex XN-1000V	1.778	(-16.846, 20.401)	0.851
		37	Sysmex XN-1000V	19.472	(0.849, 38.096)	0.040
	48	4	Sysmex XN-1000V	8.806	(-9.818, 27.429)	0.353
		20	Sysmex XN-1000V	-6.917	(-25.540, 11.707)	0.466
		30	Sysmex XN-1000V	12.821	(-5.891, 31.534)	0.179
		37	Sysmex XN-1000V	16.222	(-2.401, 34.846)	0.088
	72	4	Sysmex XN-1000V	18.389	(-0.235, 37.013)	0.053

Analyzer	Time	Temperature	Instrument	Difference	95% CI	p-value
		20	Sysmex XN-1000V	-2.750	(-21.374, 15.874)	0.772
		30	Sysmex XN-1000V	10.528	(-8.096, 29.151)	0.267
		37	Sysmex XN-1000V	22.417	(3.793, 41.040)	0.018

Greyed values indicate difference of statistical significance ($p<0.05$) from baseline (3h)

B Neutrophils (cellsx10^9/L)

Analyzer	Time	Temperature	Instrument	Difference	95% CI	p-value
Siemens Advia 2120i	6	4	Siemens Advia 2120i	-0.051	(-0.242, 0.139)	0.597
		20	Siemens Advia 2120i	0.005	(-0.186, 0.196)	0.959
		30	Siemens Advia 2120i	0.000	(-0.191, 0.191)	1.000
		37	Siemens Advia 2120i	0.051	(-0.140, 0.242)	0.599
	24	4	Siemens Advia 2120i	0.047	(-0.144, 0.238)	0.629
		20	Siemens Advia 2120i	-0.135	(-0.326, 0.056)	0.165
		30	Siemens Advia 2120i	0.104	(-0.087, 0.295)	0.284
		37	Siemens Advia 2120i	0.310	(0.119, 0.501)	0.002
	48	4	Siemens Advia 2120i	0.759	(0.568, 0.949)	<.001
		20	Siemens Advia 2120i	-0.141	(-0.332, 0.050)	0.148
		30	Siemens Advia 2120i	0.136	(-0.055, 0.327)	0.162
		37	Siemens Advia 2120i	0.503	(0.312, 0.694)	<.001
	72	4	Siemens Advia 2120i	1.126	(0.935, 1.316)	<.001
		20	Siemens Advia 2120i	-0.129	(-0.320, 0.062)	0.185
		30	Siemens Advia 2120i	0.200	(0.009, 0.391)	0.040
		37	Siemens Advia 2120i	0.824	(0.634, 1.015)	<.001
Beckman Coulter DxH900	6	4	Beckman Coulter DxH900	0.047	(-0.191, 0.285)	0.698
		20	Beckman Coulter DxH900	0.122	(-0.116, 0.360)	0.313
		30	Beckman Coulter DxH900	0.093	(-0.144, 0.331)	0.441
		37	Beckman Coulter DxH900	0.131	(-0.107, 0.368)	0.281
	24	4	Beckman Coulter DxH900	0.130	(-0.108, 0.367)	0.284
		20	Beckman Coulter DxH900	0.006	(-0.232, 0.244)	0.961
		30	Beckman Coulter DxH900	-0.088	(-0.325, 0.150)	0.469
		37	Beckman Coulter DxH900	-0.221	(-0.458, 0.017)	0.069
	48	4	Beckman Coulter DxH900	0.931	(0.693, 1.169)	<.001
		20	Beckman Coulter DxH900	-0.337	(-0.574, -0.099)	0.006
		30	Beckman Coulter DxH900	-0.479	(-0.718, -0.240)	<.001
		37	Beckman Coulter DxH900	-0.756	(-0.994, -0.519)	<.001

Analyzer	Time	Temperature	Instrument	Difference	95% CI	p-value
	72	4	Beckman Coulter DxH900	1.363	(1.125, 1.600)	<.001
		20	Beckman Coulter DxH900	-0.642	(-0.880, -0.405)	<.001
		30	Beckman Coulter DxH900	-0.644	(-0.882, -0.407)	<.001
		37	Beckman Coulter DxH900	-1.173	(-1.410, -0.935)	<.001
Abbott CELL-DYN Sapphire	6	4	Abbott CELL-DYN Sapphire	-0.048	(-0.233, 0.138)	0.613
		20	Abbott CELL-DYN Sapphire	-0.041	(-0.226, 0.145)	0.663
		30	Abbott CELL-DYN Sapphire	-0.051	(-0.236, 0.135)	0.588
		37	Abbott CELL-DYN Sapphire	0.033	(-0.152, 0.219)	0.722
	24	4	Abbott CELL-DYN Sapphire	-0.037	(-0.223, 0.148)	0.689
		20	Abbott CELL-DYN Sapphire	-0.182	(-0.367, 0.004)	0.055
		30	Abbott CELL-DYN Sapphire	-0.164	(-0.350, 0.021)	0.082
		37	Abbott CELL-DYN Sapphire	-0.074	(-0.260, 0.111)	0.430
	48	4	Abbott CELL-DYN Sapphire	0.201	(0.000, 0.401)	0.050
		20	Abbott CELL-DYN Sapphire	-0.340	(-0.541, -0.140)	0.001
		30	Abbott CELL-DYN Sapphire	-0.279	(-0.480, -0.079)	0.007
		37	Abbott CELL-DYN Sapphire	-0.457	(-0.658, -0.256)	<.001
Sysmex XN-1000V	6	4	Sysmex XN-1000V	0.013	(-0.261, 0.287)	0.925
		20	Sysmex XN-1000V	0.011	(-0.263, 0.285)	0.935
		30	Sysmex XN-1000V	0.059	(-0.214, 0.333)	0.670
		37	Sysmex XN-1000V	-0.035	(-0.309, 0.239)	0.800
	24	4	Sysmex XN-1000V	0.134	(-0.140, 0.408)	0.337
		20	Sysmex XN-1000V	-0.083	(-0.357, 0.191)	0.550
		30	Sysmex XN-1000V	-0.038	(-0.312, 0.236)	0.785
		37	Sysmex XN-1000V	-0.138	(-0.412, 0.136)	0.324
	48	4	Sysmex XN-1000V	0.809	(0.535, 1.083)	<.001
		20	Sysmex XN-1000V	-0.176	(-0.450, 0.098)	0.207
		30	Sysmex XN-1000V	-0.362	(-0.637, -0.087)	0.010
		37	Sysmex XN-1000V	-0.513	(-0.787, -0.239)	<.001
	72	4	Sysmex XN-1000V	1.295	(1.019, 1.570)	<.001

Analyzer	Time	Temperature	Instrument	Difference	95% CI	p-value
		20	Sysmex XN-1000V	-0.402	(-0.676, -0.128)	0.004
		30	Sysmex XN-1000V	-0.544	(-0.818, -0.270)	<.001
		37	Sysmex XN-1000V	-0.122	(-0.396, 0.152)	0.382

Greyed values indicate difference of statistical significance (p<0.05) from baseline (3h)

C Lymphocytes

Analyzer	Time	Temperature	Instrument	Difference	95% CI	p-value
Siemens Advia 2120i	6	4	Siemens Advia 2120i	0.006	(-0.056, 0.067)	0.859
		20	Siemens Advia 2120i	0.019	(-0.043, 0.080)	0.551
		30	Siemens Advia 2120i	0.051	(-0.011, 0.112)	0.106
		37	Siemens Advia 2120i	0.077	(0.016, 0.138)	0.014
	24	4	Siemens Advia 2120i	0.098	(0.037, 0.159)	0.002
		20	Siemens Advia 2120i	0.089	(0.027, 0.150)	0.005
		30	Siemens Advia 2120i	0.173	(0.111, 0.234)	<.001
		37	Siemens Advia 2120i	0.357	(0.296, 0.418)	<.001
	48	4	Siemens Advia 2120i	0.213	(0.152, 0.275)	<.001
		20	Siemens Advia 2120i	0.182	(0.121, 0.244)	<.001
		30	Siemens Advia 2120i	0.375	(0.313, 0.436)	<.001
		37	Siemens Advia 2120i	0.568	(0.507, 0.629)	<.001
	72	4	Siemens Advia 2120i	0.245	(0.184, 0.307)	<.001
		20	Siemens Advia 2120i	0.276	(0.215, 0.337)	<.001
		30	Siemens Advia 2120i	0.485	(0.423, 0.546)	<.001
		37	Siemens Advia 2120i	0.680	(0.618, 0.741)	<.001
Beckman Coulter DxH900	6	4	Beckman Coulter DxH900	-0.048	(-0.207, 0.111)	0.557
		20	Beckman Coulter DxH900	-0.051	(-0.211, 0.108)	0.526
		30	Beckman Coulter DxH900	-0.050	(-0.210, 0.109)	0.533
		37	Beckman Coulter DxH900	-0.066	(-0.225, 0.093)	0.416
	24	4	Beckman Coulter DxH900	-0.193	(-0.352, -0.034)	0.018
		20	Beckman Coulter DxH900	-0.119	(-0.278, 0.040)	0.142
		30	Beckman Coulter DxH900	0.040	(-0.119, 0.199)	0.619
		37	Beckman Coulter DxH900	0.229	(0.070, 0.388)	0.005
	48	4	Beckman Coulter DxH900	-0.288	(-0.447, -0.129)	<.001
		20	Beckman Coulter DxH900	0.059	(-0.100, 0.218)	0.468
		30	Beckman Coulter DxH900	0.323	(0.163, 0.483)	<.001
		37	Beckman Coulter DxH900	1.039	(0.880, 1.198)	<.001

Analyzer	Time	Temperature	Instrument	Difference	95% CI	p-value
	72	4	Beckman Coulter DxH900	-0.263	(-0.422, -0.103)	0.001
		20	Beckman Coulter DxH900	0.284	(0.125, 0.444)	<.001
		30	Beckman Coulter DxH900	0.633	(0.474, 0.792)	<.001
		37	Beckman Coulter DxH900	1.236	(1.076, 1.395)	<.001
Abbott CELL-DYN Sapphire	6	4	Abbott CELL-DYN Sapphire	0.036	(-0.126, 0.199)	0.661
		20	Abbott CELL-DYN Sapphire	-0.050	(-0.213, 0.113)	0.544
		30	Abbott CELL-DYN Sapphire	-0.025	(-0.188, 0.138)	0.762
		37	Abbott CELL-DYN Sapphire	0.085	(-0.078, 0.248)	0.303
	24	4	Abbott CELL-DYN Sapphire	0.060	(-0.102, 0.223)	0.465
		20	Abbott CELL-DYN Sapphire	0.061	(-0.102, 0.223)	0.461
		30	Abbott CELL-DYN Sapphire	0.085	(-0.078, 0.248)	0.303
		37	Abbott CELL-DYN Sapphire	0.046	(-0.117, 0.208)	0.580
	48	4	Abbott CELL-DYN Sapphire	0.146	(-0.030, 0.322)	0.103
		20	Abbott CELL-DYN Sapphire	0.068	(-0.108, 0.244)	0.446
		30	Abbott CELL-DYN Sapphire	0.001	(-0.174, 0.177)	0.988
		37	Abbott CELL-DYN Sapphire	0.502	(0.326, 0.677)	<.001
Sysmex XN-1000V	6	4	Sysmex XN-1000V	0.034	(-0.132, 0.201)	0.685
		20	Sysmex XN-1000V	-0.001	(-0.168, 0.165)	0.987
		30	Sysmex XN-1000V	0.019	(-0.148, 0.186)	0.824
		37	Sysmex XN-1000V	0.008	(-0.159, 0.175)	0.927
	24	4	Sysmex XN-1000V	0.041	(-0.126, 0.208)	0.631
		20	Sysmex XN-1000V	0.026	(-0.141, 0.193)	0.761
		30	Sysmex XN-1000V	0.032	(-0.135, 0.198)	0.709
		37	Sysmex XN-1000V	0.323	(0.156, 0.490)	<.001
	48	4	Sysmex XN-1000V	0.054	(-0.112, 0.221)	0.522
		20	Sysmex XN-1000V	0.061	(-0.106, 0.228)	0.474
		30	Sysmex XN-1000V	0.374	(0.206, 0.541)	<.001
		37	Sysmex XN-1000V	1.041	(0.875, 1.208)	<.001
	72	4	Sysmex XN-1000V	0.117	(-0.050, 0.285)	0.170

Analyzer	Time	Temperature	Instrument	Difference	95% CI	p-value
		20	Sysmex XN-1000V	0.159	(-0.008, 0.326)	0.061
		30	Sysmex XN-1000V	0.821	(0.654, 0.988)	<.001
		37	Sysmex XN-1000V	1.303	(1.137, 1.470)	<.001

Greyed values indicate difference of statistical significance ($p<0.05$) from baseline (3h)

D Eosinophils

Analyzer	Time	Temperature	Instrument	Difference	95% CI	p-value
Siemens Advia 2120i	6	4	Siemens Advia 2120i	-0.018	(-0.061, 0.025)	0.414
		20	Siemens Advia 2120i	-0.003	(-0.046, 0.040)	0.888
		30	Siemens Advia 2120i	-0.004	(-0.047, 0.038)	0.838
		37	Siemens Advia 2120i	0.009	(-0.034, 0.052)	0.683
	24	4	Siemens Advia 2120i	-0.047	(-0.090, -0.004)	0.031
		20	Siemens Advia 2120i	0.086	(0.043, 0.129)	<.001
		30	Siemens Advia 2120i	0.125	(0.083, 0.168)	<.001
		37	Siemens Advia 2120i	0.074	(0.031, 0.116)	<.001
	48	4	Siemens Advia 2120i	-0.080	(-0.122, -0.037)	<.001
		20	Siemens Advia 2120i	0.151	(0.108, 0.194)	<.001
		30	Siemens Advia 2120i	0.136	(0.093, 0.179)	<.001
		37	Siemens Advia 2120i	0.033	(-0.009, 0.076)	0.126
	72	4	Siemens Advia 2120i	-0.041	(-0.084, 0.001)	0.058
		20	Siemens Advia 2120i	0.153	(0.110, 0.195)	<.001
		30	Siemens Advia 2120i	0.089	(0.046, 0.132)	<.001
		37	Siemens Advia 2120i	-0.029	(-0.072, 0.014)	0.185
Beckman Coulter DxH900	6	4	Beckman Coulter DxH900	0.007	(-0.055, 0.068)	0.833
		20	Beckman Coulter DxH900	0.018	(-0.043, 0.080)	0.557
		30	Beckman Coulter DxH900	0.006	(-0.055, 0.068)	0.843
		37	Beckman Coulter DxH900	0.016	(-0.046, 0.077)	0.619
	24	4	Beckman Coulter DxH900	-0.031	(-0.093, 0.030)	0.320
		20	Beckman Coulter DxH900	0.022	(-0.039, 0.084)	0.477
		30	Beckman Coulter DxH900	-0.008	(-0.070, 0.053)	0.795
		37	Beckman Coulter DxH900	0.014	(-0.047, 0.076)	0.652
	48	4	Beckman Coulter DxH900	-0.065	(-0.127, -0.004)	0.037
		20	Beckman Coulter DxH900	0.028	(-0.034, 0.089)	0.378
		30	Beckman Coulter DxH900	-0.008	(-0.070, 0.054)	0.799
		37	Beckman Coulter DxH900	-0.081	(-0.143, -0.020)	0.010

Analyzer	Time	Temperature	Instrument	Difference	95% CI	p-value
	72	4	Beckman Coulter DxH900	-0.091	(-0.153, -0.030)	0.004
		20	Beckman Coulter DxH900	-0.018	(-0.079, 0.044)	0.575
		30	Beckman Coulter DxH900	-0.069	(-0.130, -0.007)	0.029
		37	Beckman Coulter DxH900	-0.119	(-0.181, -0.058)	<.001
Abbott CELL-DYN Sapphire	6	4	Abbott CELL-DYN Sapphire	0.017	(-0.089, 0.122)	0.755
		20	Abbott CELL-DYN Sapphire	0.021	(-0.085, 0.126)	0.698
		30	Abbott CELL-DYN Sapphire	0.014	(-0.091, 0.119)	0.790
		37	Abbott CELL-DYN Sapphire	0.050	(-0.056, 0.155)	0.353
	24	4	Abbott CELL-DYN Sapphire	-0.003	(-0.108, 0.102)	0.953
		20	Abbott CELL-DYN Sapphire	0.156	(0.051, 0.261)	0.004
		30	Abbott CELL-DYN Sapphire	0.181	(0.075, 0.286)	<.001
		37	Abbott CELL-DYN Sapphire	0.206	(0.100, 0.311)	<.001
	48	4	Abbott CELL-DYN Sapphire	-0.003	(-0.117, 0.111)	0.960
		20	Abbott CELL-DYN Sapphire	0.285	(0.171, 0.399)	<.001
		30	Abbott CELL-DYN Sapphire	0.306	(0.193, 0.420)	<.001
		37	Abbott CELL-DYN Sapphire	0.294	(0.181, 0.408)	<.001
Sysmex XN-1000V	6	4	Sysmex XN-1000V	0.004	(-0.138, 0.146)	0.957
		20	Sysmex XN-1000V	-0.003	(-0.145, 0.139)	0.966
		30	Sysmex XN-1000V	0.003	(-0.139, 0.145)	0.966
		37	Sysmex XN-1000V	-0.006	(-0.149, 0.136)	0.930
	24	4	Sysmex XN-1000V	0.010	(-0.132, 0.152)	0.887
		20	Sysmex XN-1000V	-0.009	(-0.151, 0.133)	0.902
		30	Sysmex XN-1000V	-0.009	(-0.151, 0.134)	0.905
		37	Sysmex XN-1000V	0.004	(-0.138, 0.147)	0.951
	48	4	Sysmex XN-1000V	-0.011	(-0.153, 0.132)	0.884
		20	Sysmex XN-1000V	-0.017	(-0.159, 0.125)	0.812
		30	Sysmex XN-1000V	-0.127	(-0.270, 0.016)	0.081
		37	Sysmex XN-1000V	-0.044	(-0.186, 0.098)	0.544
	72	4	Sysmex XN-1000V	-0.008	(-0.151, 0.135)	0.911

Analyzer	Time	Temperature	Instrument	Difference	95% CI	p-value
		20	Sysmex XN-1000V	0.018	(-0.124, 0.160)	0.800
		30	Sysmex XN-1000V	-0.092	(-0.234, 0.050)	0.204
		37	Sysmex XN-1000V	0.032	(-0.110, 0.174)	0.656

Greyed values indicate difference of statistical significance (p<0.05) from baseline (3h)

E Monocytes

Analyzer	Time	Temperature	Instrument	Difference	95% CI	p-value
Siemens Advia 2120i	6	4	Siemens Advia 2120i	0.015	(-0.093, 0.123)	0.784
		20	Siemens Advia 2120i	0.002	(-0.106, 0.109)	0.972
		30	Siemens Advia 2120i	-0.001	(-0.108, 0.107)	0.992
		37	Siemens Advia 2120i	0.013	(-0.094, 0.121)	0.812
	24	4	Siemens Advia 2120i	-0.036	(-0.144, 0.071)	0.506
		20	Siemens Advia 2120i	-0.035	(-0.143, 0.072)	0.519
		30	Siemens Advia 2120i	-0.120	(-0.227, -0.012)	0.029
		37	Siemens Advia 2120i	-0.032	(-0.139, 0.076)	0.563
	48	4	Siemens Advia 2120i	-0.055	(-0.163, 0.053)	0.315
		20	Siemens Advia 2120i	-0.024	(-0.131, 0.084)	0.663
		30	Siemens Advia 2120i	-0.110	(-0.218, -0.003)	0.044
		37	Siemens Advia 2120i	-0.083	(-0.191, 0.024)	0.128
	72	4	Siemens Advia 2120i	-0.051	(-0.159, 0.056)	0.348
		20	Siemens Advia 2120i	-0.032	(-0.140, 0.075)	0.556
		30	Siemens Advia 2120i	-0.104	(-0.212, 0.003)	0.057
		37	Siemens Advia 2120i	-0.188	(-0.296, -0.081)	<.001
Beckman Coulter DxH900	6	4	Beckman Coulter DxH900	-0.012	(-0.077, 0.053)	0.720
		20	Beckman Coulter DxH900	-0.003	(-0.067, 0.062)	0.937
		30	Beckman Coulter DxH900	-0.001	(-0.065, 0.064)	0.983
		37	Beckman Coulter DxH900	-0.008	(-0.073, 0.056)	0.800
	24	4	Beckman Coulter DxH900	-0.061	(-0.126, 0.004)	0.064
		20	Beckman Coulter DxH900	0.053	(-0.011, 0.118)	0.107
		30	Beckman Coulter DxH900	0.142	(0.077, 0.207)	<.001
		37	Beckman Coulter DxH900	0.267	(0.202, 0.331)	<.001
	48	4	Beckman Coulter DxH900	-0.108	(-0.173, -0.043)	0.001
		20	Beckman Coulter DxH900	0.171	(0.106, 0.235)	<.001
		30	Beckman Coulter DxH900	0.317	(0.252, 0.382)	<.001
		37	Beckman Coulter DxH900	0.184	(0.120, 0.249)	<.001

Analyzer	Time	Temperature	Instrument	Difference	95% CI	p-value
	72	4	Beckman Coulter DxH900	-0.086	(-0.151, -0.022)	0.009
		20	Beckman Coulter DxH900	0.287	(0.222, 0.351)	<.001
		30	Beckman Coulter DxH900	0.315	(0.250, 0.379)	<.001
		37	Beckman Coulter DxH900	0.294	(0.230, 0.359)	<.001
Abbott CELL-DYN Sapphire	6	4	Abbott CELL-DYN Sapphire	0.004	(-0.063, 0.071)	0.908
		20	Abbott CELL-DYN Sapphire	-0.031	(-0.097, 0.036)	0.367
		30	Abbott CELL-DYN Sapphire	-0.003	(-0.070, 0.064)	0.922
		37	Abbott CELL-DYN Sapphire	0.017	(-0.049, 0.084)	0.605
	24	4	Abbott CELL-DYN Sapphire	-0.026	(-0.093, 0.041)	0.446
		20	Abbott CELL-DYN Sapphire	-0.034	(-0.101, 0.033)	0.312
		30	Abbott CELL-DYN Sapphire	0.046	(-0.021, 0.113)	0.173
		37	Abbott CELL-DYN Sapphire	0.074	(0.007, 0.141)	0.031
	48	4	Abbott CELL-DYN Sapphire	0.054	(-0.018, 0.126)	0.143
		20	Abbott CELL-DYN Sapphire	-0.019	(-0.091, 0.054)	0.607
		30	Abbott CELL-DYN Sapphire	0.238	(0.166, 0.311)	<.001
		37	Abbott CELL-DYN Sapphire	0.183	(0.111, 0.256)	<.001
Sysmex XN-1000V	6	4	Sysmex XN-1000V	-0.011	(-0.128, 0.106)	0.855
		20	Sysmex XN-1000V	-0.017	(-0.134, 0.099)	0.769
		30	Sysmex XN-1000V	0.002	(-0.115, 0.119)	0.970
		37	Sysmex XN-1000V	-0.022	(-0.139, 0.095)	0.712
	24	4	Sysmex XN-1000V	-0.034	(-0.151, 0.082)	0.563
		20	Sysmex XN-1000V	0.028	(-0.089, 0.144)	0.644
		30	Sysmex XN-1000V	0.059	(-0.058, 0.176)	0.320
		37	Sysmex XN-1000V	0.136	(0.019, 0.252)	0.023
	48	4	Sysmex XN-1000V	0.004	(-0.113, 0.121)	0.948
		20	Sysmex XN-1000V	0.114	(-0.003, 0.231)	0.055
		30	Sysmex XN-1000V	0.281	(0.164, 0.399)	<.001
		37	Sysmex XN-1000V	0.109	(-0.007, 0.226)	0.066
	72	4	Sysmex XN-1000V	0.047	(-0.070, 0.164)	0.430

Analyzer	Time	Temperature	Instrument	Difference	95% CI	p-value
		20	Sysmex XN-1000V	0.259	(0.142, 0.376)	<.001
		30	Sysmex XN-1000V	0.135	(0.019, 0.252)	0.023
		37	Sysmex XN-1000V	0.046	(-0.070, 0.163)	0.436

Greyed values indicate difference of statistical significance (p<0.05) from baseline (3h)

F Basophils

Analyzer	Time	Temperature	Instrument	Difference	95% CI	p-value
Siemens Advia 2120i	6	4	Siemens Advia 2120i	0.006	(-0.097, 0.108)	0.915
		20	Siemens Advia 2120i	0.002	(-0.101, 0.104)	0.970
		30	Siemens Advia 2120i	-0.006	(-0.108, 0.097)	0.911
		37	Siemens Advia 2120i	-0.032	(-0.134, 0.071)	0.541
	24	4	Siemens Advia 2120i	-0.035	(-0.138, 0.067)	0.500
		20	Siemens Advia 2120i	-0.003	(-0.105, 0.100)	0.958
		30	Siemens Advia 2120i	-0.008	(-0.110, 0.095)	0.882
		37	Siemens Advia 2120i	-0.062	(-0.165, 0.040)	0.234
	48	4	Siemens Advia 2120i	-0.109	(-0.212, -0.007)	0.037
		20	Siemens Advia 2120i	-0.007	(-0.109, 0.096)	0.894
		30	Siemens Advia 2120i	-0.065	(-0.168, 0.038)	0.214
		37	Siemens Advia 2120i	-1.053	(-1.155, -0.950)	<.001
	72	4	Siemens Advia 2120i	-0.125	(-0.228, -0.023)	0.017
		20	Siemens Advia 2120i	-0.022	(-0.125, 0.080)	0.667
		30	Siemens Advia 2120i	-0.520	(-0.622, -0.417)	<.001
		37	Siemens Advia 2120i	-1.704	(-1.806, -1.601)	<.001
Beckman Coulter DxH900	6	4	Beckman Coulter DxH900	0.010	(-0.005, 0.025)	0.196
		20	Beckman Coulter DxH900	0.029	(0.014, 0.044)	<.001
		30	Beckman Coulter DxH900	0.022	(0.007, 0.037)	0.003
		37	Beckman Coulter DxH900	0.024	(0.009, 0.039)	0.002
	24	4	Beckman Coulter DxH900	0.012	(-0.002, 0.027)	0.102
		20	Beckman Coulter DxH900	0.022	(0.007, 0.037)	0.004
		30	Beckman Coulter DxH900	0.031	(0.016, 0.046)	<.001
		37	Beckman Coulter DxH900	0.029	(0.015, 0.044)	<.001
	48	4	Beckman Coulter DxH900	0.026	(0.011, 0.041)	<.001
		20	Beckman Coulter DxH900	0.035	(0.020, 0.050)	<.001
		30	Beckman Coulter DxH900	0.032	(0.017, 0.047)	<.001
		37	Beckman Coulter DxH900	0.052	(0.037, 0.067)	<.001

Analyzer	Time	Temperature	Instrument	Difference	95% CI	p-value
	72	4	Beckman Coulter DxH900	0.032	(0.017, 0.047)	<.001
		20	Beckman Coulter DxH900	0.037	(0.022, 0.052)	<.001
		30	Beckman Coulter DxH900	0.045	(0.031, 0.060)	<.001
		37	Beckman Coulter DxH900	0.060	(0.045, 0.075)	<.001
Abbott CELL-DYN Sapphire	6	4	Abbott CELL-DYN Sapphire	-0.007	(-0.045, 0.031)	0.727
		20	Abbott CELL-DYN Sapphire	-0.029	(-0.068, 0.009)	0.130
		30	Abbott CELL-DYN Sapphire	-0.009	(-0.047, 0.029)	0.635
		37	Abbott CELL-DYN Sapphire	-0.002	(-0.040, 0.036)	0.907
	24	4	Abbott CELL-DYN Sapphire	0.023	(-0.015, 0.062)	0.225
		20	Abbott CELL-DYN Sapphire	0.003	(-0.035, 0.041)	0.883
		30	Abbott CELL-DYN Sapphire	-0.007	(-0.045, 0.032)	0.733
		37	Abbott CELL-DYN Sapphire	0.007	(-0.031, 0.045)	0.724
	48	4	Abbott CELL-DYN Sapphire	-0.005	(-0.046, 0.036)	0.814
		20	Abbott CELL-DYN Sapphire	0.001	(-0.040, 0.042)	0.954
		30	Abbott CELL-DYN Sapphire	0.020	(-0.021, 0.061)	0.339
		37	Abbott CELL-DYN Sapphire	0.042	(0.000, 0.083)	0.048
Sysmex XN-1000V	6	4	Sysmex XN-1000V	-0.003	(-0.025, 0.019)	0.805
		20	Sysmex XN-1000V	-0.003	(-0.025, 0.019)	0.786
		30	Sysmex XN-1000V	-0.001	(-0.024, 0.021)	0.902
		37	Sysmex XN-1000V	-0.003	(-0.025, 0.019)	0.786
	24	4	Sysmex XN-1000V	-0.008	(-0.030, 0.014)	0.475
		20	Sysmex XN-1000V	-0.004	(-0.026, 0.018)	0.712
		30	Sysmex XN-1000V	-0.003	(-0.025, 0.019)	0.786
		37	Sysmex XN-1000V	-0.013	(-0.035, 0.009)	0.237
	48	4	Sysmex XN-1000V	-0.017	(-0.039, 0.005)	0.140
		20	Sysmex XN-1000V	-0.004	(-0.027, 0.018)	0.694
		30	Sysmex XN-1000V	-0.003	(-0.025, 0.019)	0.784
		37	Sysmex XN-1000V	-0.102	(-0.124, -0.080)	<.001
	72	4	Sysmex XN-1000V	-0.016	(-0.038, 0.006)	0.159

Analyzer	Time	Temperature	Instrument	Difference	95% CI	p-value
		20	Sysmex XN-1000V	-0.001	(-0.023, 0.021)	0.941
		30	Sysmex XN-1000V	-0.056	(-0.079, -0.034)	<.001
		37	Sysmex XN-1000V	-0.106	(-0.128, -0.083)	<.001

Greyed values indicate difference of statistical significance (p<0.05) from baseline (3h)
