# Supplementary material for: A comparative study of blood cell count in four automated hematology analyzers: An evaluation of the impact of preanalytical factors
Source: PLoS One. 2024 May 24;19(5):e0301845. doi: 10.1371/journal.pone.0301845 (PMC11125483; doi:10.1371/journal.pone.0301845)
Supplement: S5 Table — (PDF) [file pone.0301845.s005.pdf]

Cell type	Random Effect	Analyzer	Variance (SD)
Platelets	Between Subjects		2478.9 (49.8)
	Within Subject	Siemens Advia 2120i	222.8 (14.9)
		Beckman Coulter DxH900	200.6 (14.2)
		Abbott CELL-DYN Sapphire	1133.6 (33.7)
		Sysmex XN-1000V	1078.9 (32.8)
Neutrophils	Between Subjects		0.478 (0.691)
	Within Subject	Siemens Advia 2120i	0.113 (0.337)
		Beckman Coulter DxH900	0.176 (0.419)
		Abbott CELL-DYN Sapphire	0.052 (0.229)
		Sysmex XN-1000V	0.233 (0.483)
Lymphocytes	Between Subjects		0.199 (0.446)
	Within Subject	Siemens Advia 2120i	0.012 (0.108)
		Beckman Coulter DxH900	0.079 (0.281)
		Abbott CELL-DYN Sapphire	0.041 (0.201)
		Sysmex XN-1000V	0.086 (0.294)
Eosinophils	Between Subjects		0.021 (0.145)
	Within Subject	Siemens Advia 2120i	0.006 (0.075)
		Beckman Coulter DxH900	0.012 (0.108)
		Abbott CELL-DYN Sapphire	0.017 (0.130)
		Sysmex XN-1000V	0.063 (0.251)
Monocytes	Between Subjects		0.007 (0.082)
	Within Subject	Siemens Advia 2120i	0.036 (0.190)
		Beckman Coulter DxH900	0.013 (0.114)
		Abbott CELL-DYN Sapphire	0.007 (0.083)
		Sysmex XN-1000V	0.042 (0.206)
Basophils	Between Subjects		0.000 (0.013)
	Within Subject	Siemens Advia 2120i	0.033 (0.181)
		Beckman Coulter DxH900	0.001 (0.026)

Cell type	Random Effect	Analyzer	Variance (SD)
		Abbott CELL-DYN Sapphire	0.002 (0.047)
		Sysmex XN-1000V	0.002 (0.039)

Variance (cellsx10^9/L)^2
SD=Standard Deviation (cellsx10^9/L)
Model Random effects: Subject random intercept, Within Subject grouped by Analyzer
Model Fixed (MMRM) effects: Condition*, Analyzer*Time*Temperature
*Condition was not included when modelling basophils because it caused an infinite likelihood warning
