# Supplementary material for: A comparative study of blood cell count in four automated hematology analyzers: An evaluation of the impact of preanalytical factors
Source: PLoS One. 2024 May 24;19(5):e0301845. doi: 10.1371/journal.pone.0301845 (PMC11125483; doi:10.1371/journal.pone.0301845)
Supplement: S6 Table — (PDF) [file pone.0301845.s006.pdf]

Condition		2120i (N=18)	DxH900 (N=18)	Sapphire (N=12)	XN-1000V (N=18)
Asthmatic	n	6	6	6	6
	Mean	0.552	0.572	0.579	0.568
	SD	0.254	0.298	0.331	0.308
	Median	0.560	0.553	0.573	0.555
	Min	0.220	0.194	0.172	0.180
	Max	0.980	1.079	1.150	1.080
Healthy	n	6	6	3	6
	Mean	0.140	0.140	0.106	0.142
	SD	0.089	0.107	0.066	0.091
	Median	0.155	0.147	0.131	0.145
	Min	0.000	0.000	0.032	0.010
	Max	0.260	0.313	0.156	0.270
Healthy atopic	n	6	6	3	6
	Mean	0.202	0.190	0.232	0.208
	SD	0.057	0.050	0.126	0.052
	Median	0.210	0.197	0.191	0.225
	Min	0.130	0.131	0.132	0.140
	Max	0.270	0.240	0.373	0.270
