# Supplementary material for: A comparative study of blood cell count in four automated hematology analyzers: An evaluation of the impact of preanalytical factors
Source: PLoS One. 2024 May 24;19(5):e0301845. doi: 10.1371/journal.pone.0301845 (PMC11125483; doi:10.1371/journal.pone.0301845)
Supplement: S7 Table — (PDF) [file pone.0301845.s007.pdf]

Time	Condition		2120i	DxH900	Sapphire	XN-1000V
24	Asthmatic	n	12	12	6	12
		Mean	-0.144	-0.038	-0.229	0.034
		SD	0.084	0.083	0.176	0.040
		Median	-0.115	-0.026	-0.220	0.030
		Min	-0.320	-0.221	-0.523	-0.010
		Max	-0.050	0.069	-0.036	0.120
	Healthy	n	12	12	3	12
		Mean	-0.047	-0.021	-0.051	-0.002
		SD	0.040	0.039	0.048	0.032
		Median	-0.050	-0.006	-0.074	0.000
		Min	-0.110	-0.085	-0.083	-0.070
		Max	0.020	0.030	0.004	0.040
	Healthy atopic	n	12	12	3	12
		Mean	-0.067	-0.007	-0.116	-0.006
		SD	0.029	0.060	0.108	0.024
		Median	-0.065	-0.010	-0.055	0.000
		Min	-0.110	-0.093	-0.241	-0.040
		Max	-0.030	0.111	-0.053	0.030
48	Asthmatic	n	12	12	3	12
		Mean	-0.286	-0.020	-0.650	0.052
		SD	0.137	0.161	0.232	0.135
		Median	-0.260	0.015	-0.547	0.015
		Min	-0.560	-0.333	-0.915	-0.060
		Max	-0.050	0.219	-0.487	0.470
	Healthy	n	12	12	3	12
		Mean	-0.058	-0.033	-0.066	0.015
		SD	0.049	0.052	0.080	0.046
		Median	-0.055	-0.017	-0.104	0.005

Time	Condition		2120i	DxH900	Sapphire	XN-1000V
		Min	-0.130	-0.105	-0.121	-0.030
		Max	0.010	0.045	0.026	0.110
	Healthy atopic	n	12	12	3	12
		Mean	-0.108	-0.030	-0.180	-0.015
		SD	0.054	0.089	0.131	0.024
		Median	-0.125	-0.006	-0.106	-0.005
		Min	-0.180	-0.198	-0.331	-0.070
		Max	-0.020	0.065	-0.103	0.010
