# Supplementary material for: A comparative study of blood cell count in four automated hematology analyzers: An evaluation of the impact of preanalytical factors
Source: PLoS One. 2024 May 24;19(5):e0301845. doi: 10.1371/journal.pone.0301845 (PMC11125483; doi:10.1371/journal.pone.0301845)
Supplement: S8 Table — (PDF) [file pone.0301845.s008.pdf]

Time	Condition		2120i	DxH900	Sapphire	XN-1000V
24	Asthmatic	n	12	12	6	12
		Mean	-26.9	-10.4	-36.4	7.1
		SD	12.9	18.3	15.2	9.6
		Median	-22.4	-3.4	-42.4	6.0
		Min	-56.4	-57.0	-51.5	-2.9
		Max	-13.3	6.5	-14.3	33.3
	Healthy	n	10	10	3	12
		Mean	-34.6	-21.4	-32.8	-17.4
		SD	8.4	27.9	40.0	42.5
		Median	-35.9	-8.1	-47.4	0.0
		Min	-44.4	-66.9	-63.4	-100.0
		Max	-22.2	9.6	12.5	22.2
	Healthy atopic	n	12	12	3	12
		Mean	-32.3	-7.5	-44.5	-2.5
		SD	8.6	32.7	18.3	11.2
		Median	-32.7	-4.3	-40.2	0.0
		Min	-45.5	-66.0	-64.6	-21.4
		Max	-20.0	46.3	-28.8	13.0
48	Asthmatic	n	12	12	3	12
		Mean	-50.9	-11.8	-80.5	9.4
		SD	10.5	41.1	4.5	20.1
		Median	-54.8	3.5	-79.6	2.8
		Min	-59.1	-85.8	-85.5	-10.7
		Max	-22.7	41.6	-76.6	68.1
	Healthy	n	10	10	3	12
		Mean	-41.9	-27.8	-25.2	175.4
		SD	18.8	38.6	92.2	409.3
		Median	-38.7	-17.3	-77.6	1.9

Time	Condition		2120i	DxH900	Sapphire	XN-1000V
		Min	-72.2	-82.7	-79.4	-15.8
		Max	-15.4	26.2	81.3	1100.0
	Healthy atopic	n	12	12	3	12
		Mean	-50.5	-15.2	-74.1	-6.7
		SD	17.2	40.7	17.0	10.3
		Median	-55.3	-4.2	-78.0	-3.6
		Min	-77.3	-82.5	-88.7	-25.9
		Max	-15.4	28.4	-55.5	6.7
