# Supplementary material for: A comparative study of blood cell count in four automated hematology analyzers: An evaluation of the impact of preanalytical factors
Source: PLoS One. 2024 May 24;19(5):e0301845. doi: 10.1371/journal.pone.0301845 (PMC11125483; doi:10.1371/journal.pone.0301845)
Supplement: S9 Table — (PDF) [file pone.0301845.s009.pdf]

	Temperature (°C)		Timepoint<br>3 hrs	24 hrs	48 hrs	72 hrs
CD11b	4	n		10	12	12
		Mean		4881.7	6863.2	7789.3
		SD		1297.3	1591.4	1616.3
		Median		4972.5	6974.0	7831.0
		Min		2895	3524	4379
		Max		7143	9263	10498
	20	n	12	12	12	12
		Mean	3602.2	4312.7	4663.7	5113.9
		SD	1123.3	1385.0	1412.5	1891.1
		Median	3373.5	3783.5	4412.5	4461.0
		Min	2251	3050	1751	3173
		Max	6779	7246	7384	9656
	30	n		12	12	12
		Mean		2621.3	2074.0	1488.6
		SD		731.0	424.1	641.2
		Median		2326.0	2028.0	1507.5
		Min		1708	1197	572
		Max		3781	2733	2654
CD123	4	n		10	12	12
		Mean		250.3	259.2	294.1
		SD		154.4	185.7	166.1
		Median		212.5	220.5	232.0
		Min		102	95	147
		Max		578	764	707
	20	n	12	12	12	12
		Mean	211.5	276.8	351.7	411.5
		SD	140.6	212.7	267.5	203.7

	Temperature (°C)		Timepoint 3 hrs	24 hrs	48 hrs	72 hrs
		Median	156.5	197.0	261.0	398.0
		Min	77	111	136	151
		Max	496	810	1033	779
	30	n		12	12	12
		Mean		318.1	307.3	576.8
		SD		218.5	214.5	414.5
		Median		275.5	241.5	431.5
		Min		100	130	267
		Max		824	848	1741
CD62L	4	n		10	12	12
		Mean		3894.2	4239.9	4167.3
		SD		627.1	763.3	658.8
		Median		3789.5	4022.5	3971.5
		Min		2869	3191	3302
		Max		4776	5779	5709
	20	n	12	12	12	12
		Mean	4080.8	3518.6	3187.9	2244.7
		SD	512.7	511.0	646.5	573.6
		Median	4088.0	3643.0	3305.0	2384.5
		Min	3111	2539	1838	1497
		Max	4916	4219	4313	3083
	30	n		12	12	12
		SD		511.0	352.8	357.3
		Mean		3155.6	2467.7	1894.7
		Median		3191.5	2422.5	1974.0
		Min		2365	2023	1107
		Max		3787	3180	2349
CD66b	4	n		10	12	12

	Temperature (°C)		Timepoint 3 hrs	24 hrs	48 hrs	72 hrs
		Mean		4382.4	5344.3	5701.5
		SD		877.2	1041.5	936.8
		Median		4234.5	5158.5	5581.5
		Min		3231	3957	4287
		Max		6235	7553	7166
	20	n	12	12	12	12
		Mean	3781.8	4010.0	4161.6	4432.1
		SD	885.8	861.5	839.9	1271.5
		Median	3510.0	3970.0	4019.5	4213.5
		Min	2762	2831	3167	3177
		Max	5966	5967	5735	7218
	30	n		12	12	12
		Mean		3690.4	3542.3	3346.5
		SD		743.5	577.6	699.3
		Median		3717.5	3402.5	3089.0
		Min		2819	2794	2448
		Max		5539	4962	4608
FceR1	4	n		10	12	12
		Mean		433.7	390.9	372.3
		SD		85.4	95.3	95.3
		Median		411.5	370.5	357.5
		Min		309	244	231
		Max		576	552	524
	20	n	12	12	12	12
		Mean	430.6	412.4	404.3	444.9
		SD	106.3	96.8	88.6	95.8
		Median	409.5	390.0	387.5	474.5
		Min	274	284	249	248

	Temperature (°C)		Timepoint 3 hrs	24 hrs	48 hrs	72 hrs
		Max	603	567	536	578
	30	n		12	12	12
		Mean		411.8	395.0	542.3
		SD		91.5	86.6	151.4
		Median		389.0	370.5	525.5
		Min		278	285	374
		Max		565	542	888
LIVE-DEAD Aqua	4	n		10	12	12
		Mean		2355.8	2261.8	2187.8
		SD		286.9	253.1	246.1
		Median		2411.0	2212.0	2173.5
		Min		2037	1965	1815
		Max		2899	2819	2669
	20	n	12	12	12	12
		Mean	2192.1	2587.8	3597.2	3656.5
		SD	236.9	280.6	958.3	1333.0
		Median	2189.0	2577.0	3390.0	2975.5
		Min	1881	2182	2738	2068
		Max	2651	3117	6443	5731
	30	n		12	12	12
		Mean		2946.4	5102.8	12531.1
		SD		617.4	2613.3	13052.6
		Median		2791.0	4881.5	5321.0
		Min		2151	1084	1529
		Max		4080	11986	44909
EDN	4	n		36	36	36
		Mean		24.6	51.5	78.6
		SD		16.3	29.0	37.3

		Timepoint			
Temperature (°C)		3 hrs	24 hrs	48 hrs	72 hrs
	Median		17.3	44.0	75.8
	Min		4	14	25
	Max		77	106	150
20	n	17	36	36	36
	Mean	18.3	22.2	24.8	32.8
	SD	10.5	13.0	14.1	16.5
	Median	14.7	17.8	18.5	26.8
	Min	7	4	7	12
	Max	45	63	55	73
30	n		36	36	36
	Mean		32.0	63.0	144.4
	SD		18.1	49.8	144.3
	Median		25.6	37.3	81.8
	Min		8	16	43
	Max		72	197	571
37	n		36	36	32
	Mean		53.7	232.2	479.1
	SD		32.3	272.7	451.2
	Median		39.7	125.8	270.2
	Min		16	52	70
	Max		137	1200	1715
