# Supplementary material for: A comparative study of blood cell count in four automated hematology analyzers: An evaluation of the impact of preanalytical factors
Source: PLoS One. 2024 May 24;19(5):e0301845. doi: 10.1371/journal.pone.0301845 (PMC11125483; doi:10.1371/journal.pone.0301845)
Supplement: S10 Table — (PDF) [file pone.0301845.s010.pdf]

	Time	Temperature	n	LS Mean diff	95% CI	p-value
CD11b	24	4	12	-1274	(-2119, -429)	0.004
		20	12	-711	(-1513, 92)	0.082
		30	12	981	(179, 1783)	0.017
	48	4	12	-3261	(-4063, -2459)	<.001
		20	12	-1062	(-1864, -259)	0.010
		30	12	1528	(726, 2330)	<.001
	72	4	12	-4187	(-4989, -3385)	<.001
		20	12	-1512	(-2314, -710)	<.001
		30	12	2114	(1311, 2916)	<.001
CD123	24	4	12	-17	(-160, 126)	0.815
		20	12	-65	(-201, 71)	0.342
		30	12	-107	(-242, 29)	0.122
	48	4	12	-48	(-184, 88)	0.487
		20	12	-140	(-276, -4)	0.043
		30	12	-96	(-232, 40)	0.165
	72	4	12	-83	(-218, 53)	0.230
		20	12	-200	(-336, -64)	0.004
		30	12	-365	(-501, -229)	<.001
CD62L	24	4	12	158	(-189, 505)	0.368
		20	12	562	(233, 892)	0.001
		30	12	925	(596, 1255)	<.001
	48	4	12	-159	(-489, 170)	0.340
		20	12	893	(563, 1222)	<.001
		30	12	1613	(1284, 1943)	<.001
	72	4	12	-86	(-416, 243)	0.604
		20	12	1836	(1507, 2166)	<.001
		30	12	2186	(1857, 2516)	<.001

	Time	Temperature	n	LS Mean diff	95% CI	p-value
CD66b	24	4	12	-667	(-1084, -250)	0.002
		20	12	-228	(-624, 168)	0.255
		30	12	91	(-304, 487)	0.648
	48	4	12	-1562	(-1958, -1167)	<.001
		20	12	-380	(-776, 16)	0.060
		30	12	240	(-156, 635)	0.233
	72	4	12	-1920	(-2316, -1524)	<.001
		20	12	-650	(-1046, -254)	0.002
		30	12	435	(39, 831)	0.031
FceR1	24	4	12	9	(-54, 72)	0.782
		20	12	18	(-42, 78)	0.547
		30	12	19	(-41, 79)	0.533
	48	4	12	40	(-20, 99)	0.190
		20	12	26	(-33, 86)	0.385
		30	12	36	(-24, 95)	0.240
	72	4	12	58	(-1, 118)	0.056
		20	12	-14	(-74, 45)	0.635
		30	12	-112	(-171, -52)	<.001
LIVE-DEAD	24	4	12	-135	(-3759, 3490)	0.941
		20	12	-396	(-3845, 3054)	0.820
		30	12	-754	(-4204, 2695)	0.665
	48	4	12	-70	(-3519, 3380)	0.968
		20	12	-1405	(-4854, 2044)	0.421
		30	12	-2911	(-6360, 539)	0.097
	72	4	12	4	(-3445, 3453)	0.998
		20	12	-1464	(-4914, 1985)	0.402
		30	12	-10339	(-13788, -6890)	<.001
EDN	24	4	18	-10	(-109, 88)	0.837
		20	18	-6	(-104, 93)	0.910

Time	Temperature	n	LS Mean diff	95% CI	p-value
	30	18	-17	(-115, 82)	0.739
	37	18	-39	(-138, 60)	0.438
48	4	18	-36	(-135, 63)	0.474
	20	18	-10	(-108, 89)	0.847
	30	18	-50	(-148, 49)	0.323
	37	18	-224	(-322, -125)	<.001
72	4	18	-64	(-162, 35)	0.205
	20	18	-18	(-116, 81)	0.724
	30	18	-132	(-231, -34)	0.009
	37	18	-478	(-579, -376)	<.001

Greyed values indicate difference of statistical significance (p<0.05) from baseline (3h)
