# Supplementary material for: A comparative study of blood cell count in four automated hematology analyzers: An evaluation of the impact of preanalytical factors
Source: PLoS One. 2024 May 24;19(5):e0301845. doi: 10.1371/journal.pone.0301845 (PMC11125483; doi:10.1371/journal.pone.0301845)

A

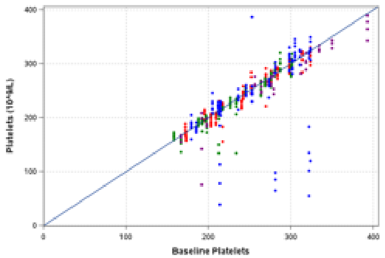

Analyzer	Spearman's Correlation r	Difference in LS means 6-3hrs (95% CI)
Advia 2120i	0.95589	0.71 (-7.74, 9.19)
Beckman DxH900	0.95188	1.07 (-6.96, 9.10)
CELL-DYN Sapphire	0.95600	3.67 (-23.54, 30.87)
Sysmex XN-1000V	0.71180	-2.11 (-20.73, 16.51)

B

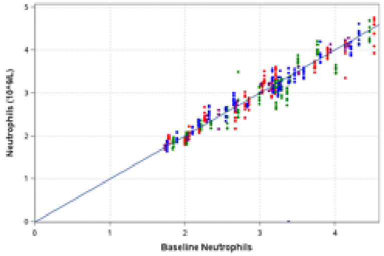

Analyzer	Spearman's Correlation r	Difference in LS means 6-3hrs (95% CI)
Advia 2120i	0.96277	0.01 (-0.19, 0.20)
Beckman DxH900	0.94187	0.12 (-0.12, 0.36)
CELL-DYN Sapphire	0.98478	-0.04 (-0.23, 0.15)
Sysmex XN-1000V	0.94265	0.01 (-0.26, 0.29)

C

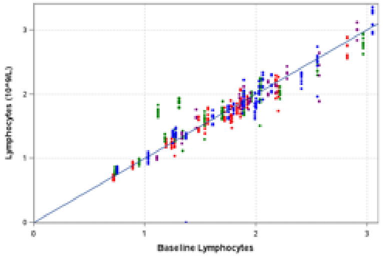

Analyzer	Spearman's Correlation r	Difference in LS means 6-3hrs (95% CI)
Advia 2120i	0.94919	0.019 (-0.043, 0.080)
Beckman DxH900	0.87880	-0.051 (-0.211, 0.108)
CELL-DYN Sapphire	0.91903	-0.050 (-0.213, 0.113)
Sysmex XN-1000V	0.95122	-0.001 (-0.168, 0.165)

D

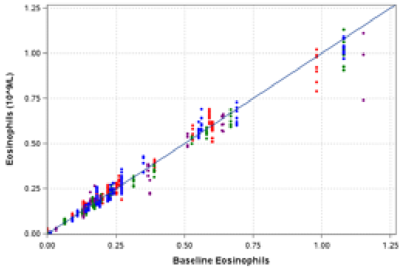

Analyzer	Spearman's Correlation r	Difference in LS means 6-3hrs (95% CI)
Advia 2120i	0.97208	-0.003 (-0.046, 0.040)
Beckman DxH900	0.98452	0.018 (-0.043, 0.080)
CELL-DYN Sapphire	0.96616	0.021 (-0.085, 0.126)
Sysmex XN-1000V	0.97322	-0.003 (-0.145, 0.139)

E

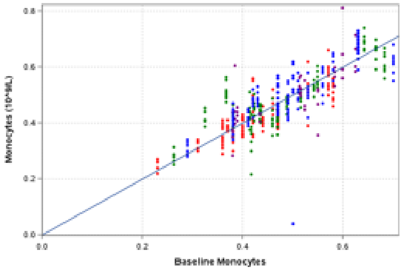

Analyzer	Spearman's Correlation r	Difference in LS means 6-3hrs (95% CI)
Advia 2120i	0.85345	0.002 (-0.101, 0.104)
Beckman DxH900	0.71884	-0.003 (-0.067, 0.062)
CELL-DYN Sapphire	0.79946	-0.031 (-0.097, 0.036)
Sysmex XN-1000V	0.84050	-0.017 (-0.134, 0.099)

F

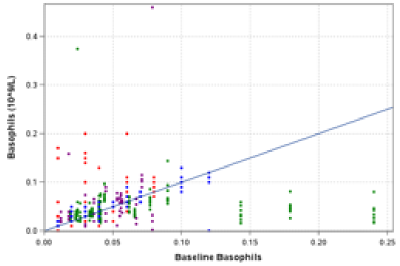

Analyzer	Spearman's Correlation r	Difference in LS means 6-3hrs (95% CI)
Advia 2120i	0.54261	0.002 (-1.101, 0.104)
Beckman DxH900	0.30963	***0.029 (0.014, 0.044)***
CELL-DYN Sapphire	0.26951	-0.029 (-0.068, 0.009)
Sysmex XN-1000V	0.84832	-0.003 (-0.025, 0.019)

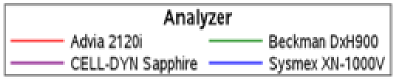

Supplement: S1 Fig — (PDF) [file pone.0301845.s011.pdf]
