# Supplementary material for: A comparative study of blood cell count in four automated hematology analyzers: An evaluation of the impact of preanalytical factors
Source: PLoS One. 2024 May 24;19(5):e0301845. doi: 10.1371/journal.pone.0301845 (PMC11125483; doi:10.1371/journal.pone.0301845)
Supplement: S2 Fig — (PDF) [file pone.0301845.s012.pdf]

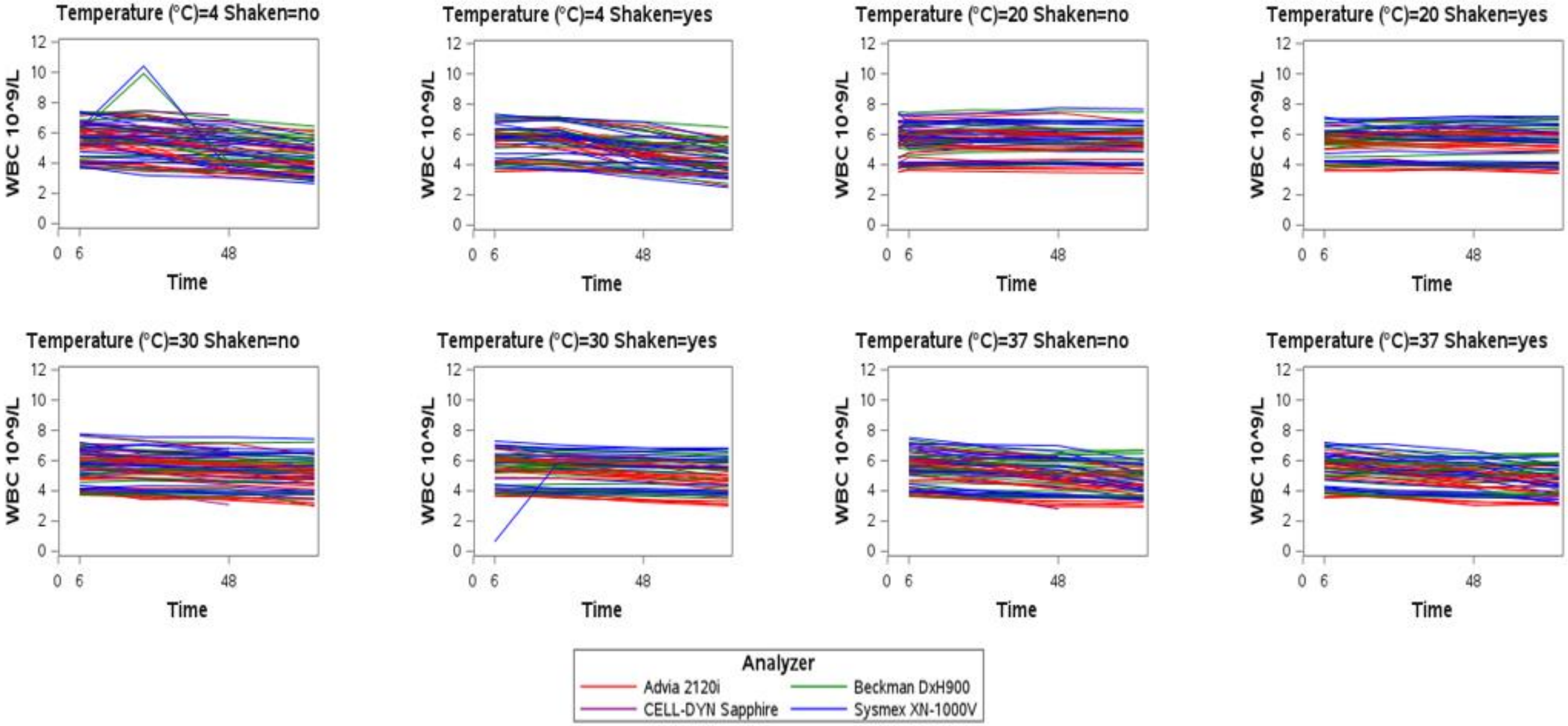

Temperature (°C)=4 Shaken=no
Temperature (°C)=4 Shaken=yes
Temperature (°C)=20 Shaken=no
Temperature (°C)=20 Shaken=yes
Temperature (°C)=30 Shaken=no
Temperature (°C)=30 Shaken=yes
Temperature (°C)=37 Shaken=no
Temperature (°C)=37 Shaken=yes
WBC 10^9/L
Time
0 6 48
0 2 4 6 8 10 12
Analyzer
Advia 2120i
Beckman DxH900
CELL-DYN Sapphire
Sysmex XN-1000V
