# Supplementary figures and images for: A comparative study of blood cell count in four automated hematology analyzers: An evaluation of the impact of preanalytical factors
Source: PLoS One. 2024 May 24;19(5):e0301845. doi: 10.1371/journal.pone.0301845 (PMC11125483; doi:10.1371/journal.pone.0301845)

A

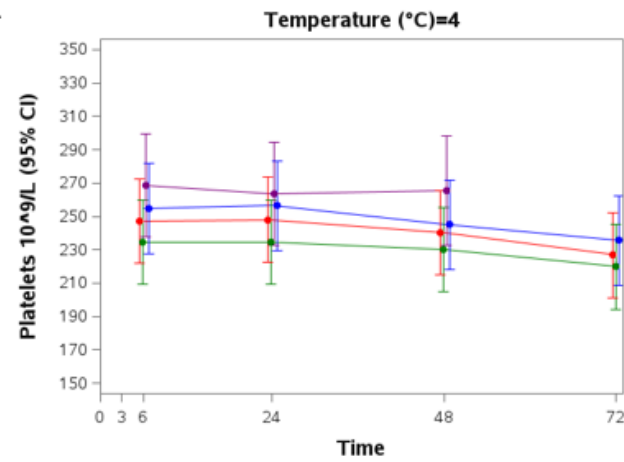

B

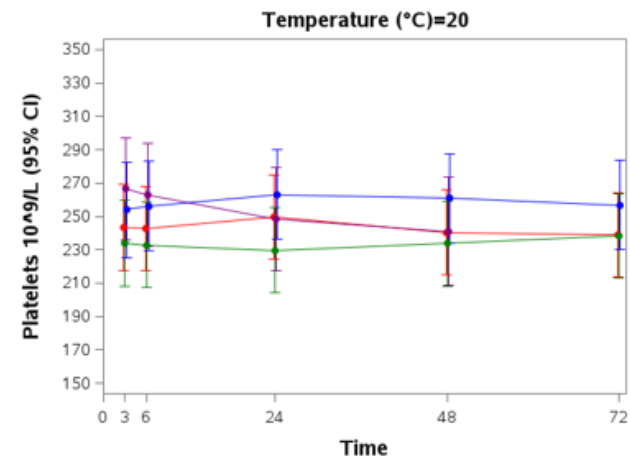

C

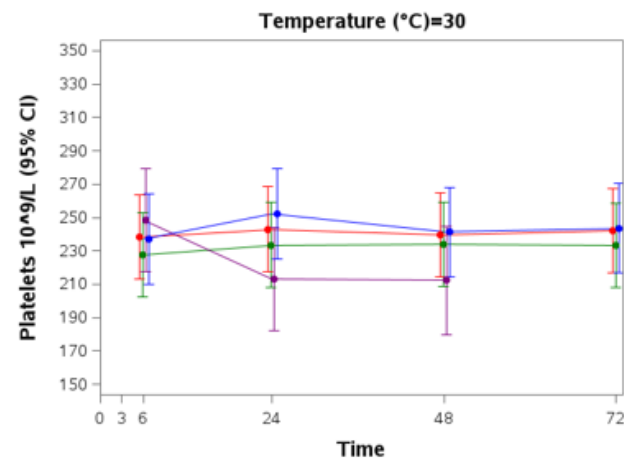

D

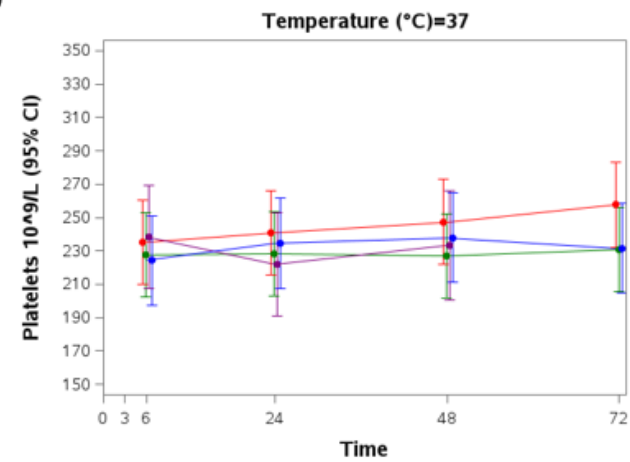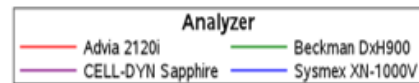

Supplement: S3 Fig — (PDF) [file pone.0301845.s013.pdf]

A

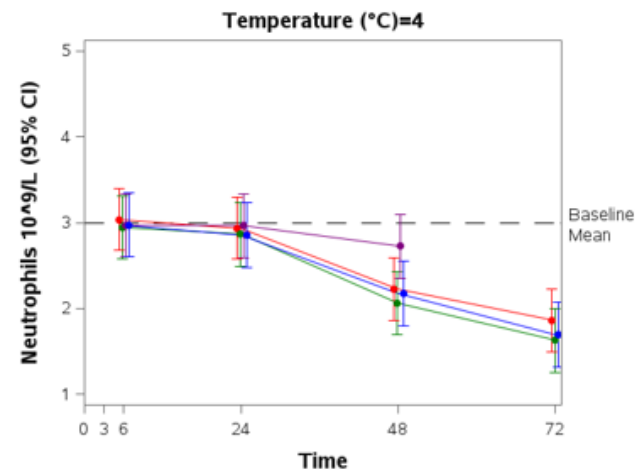

B

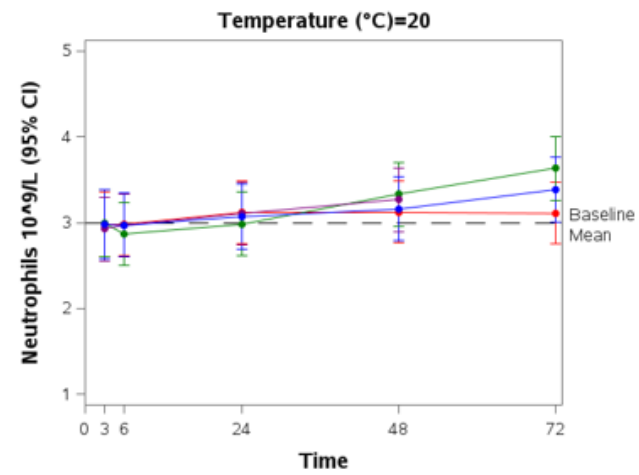

C

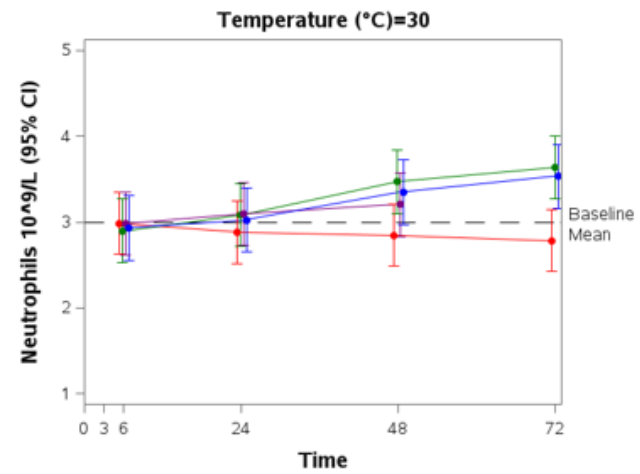

D

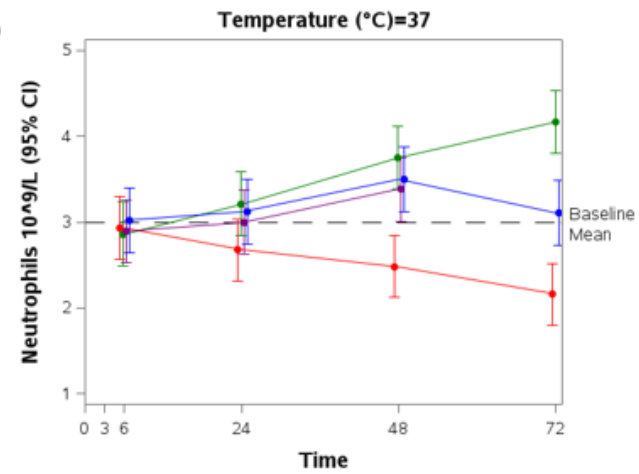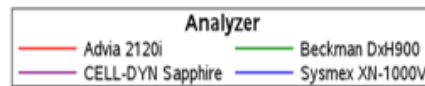

Supplement: S4 Fig — (PDF) [file pone.0301845.s014.pdf]

A

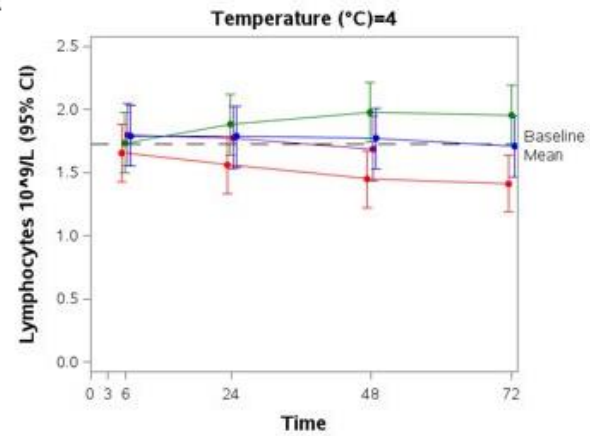

B

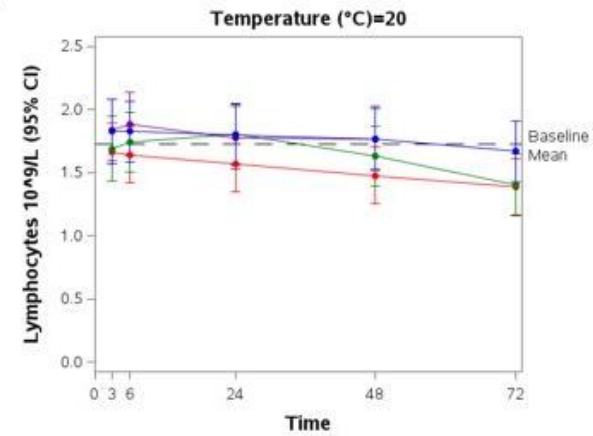

C

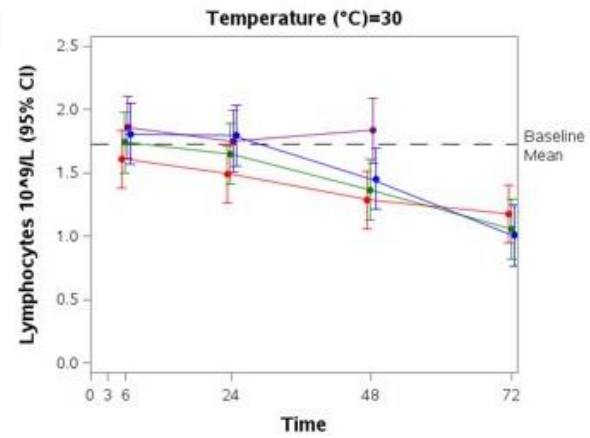

D

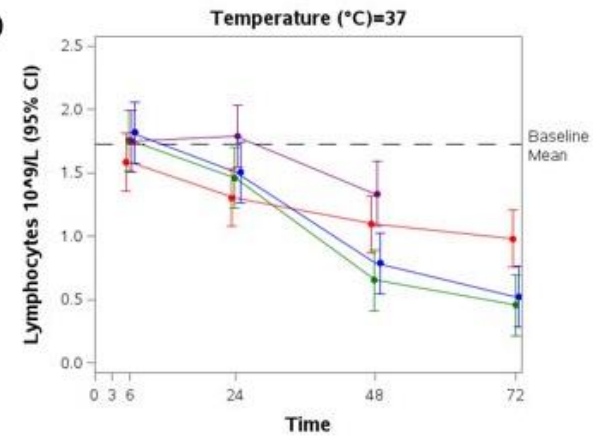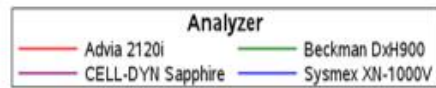

Supplement: S5 Fig — (PDF) [file pone.0301845.s015.pdf]

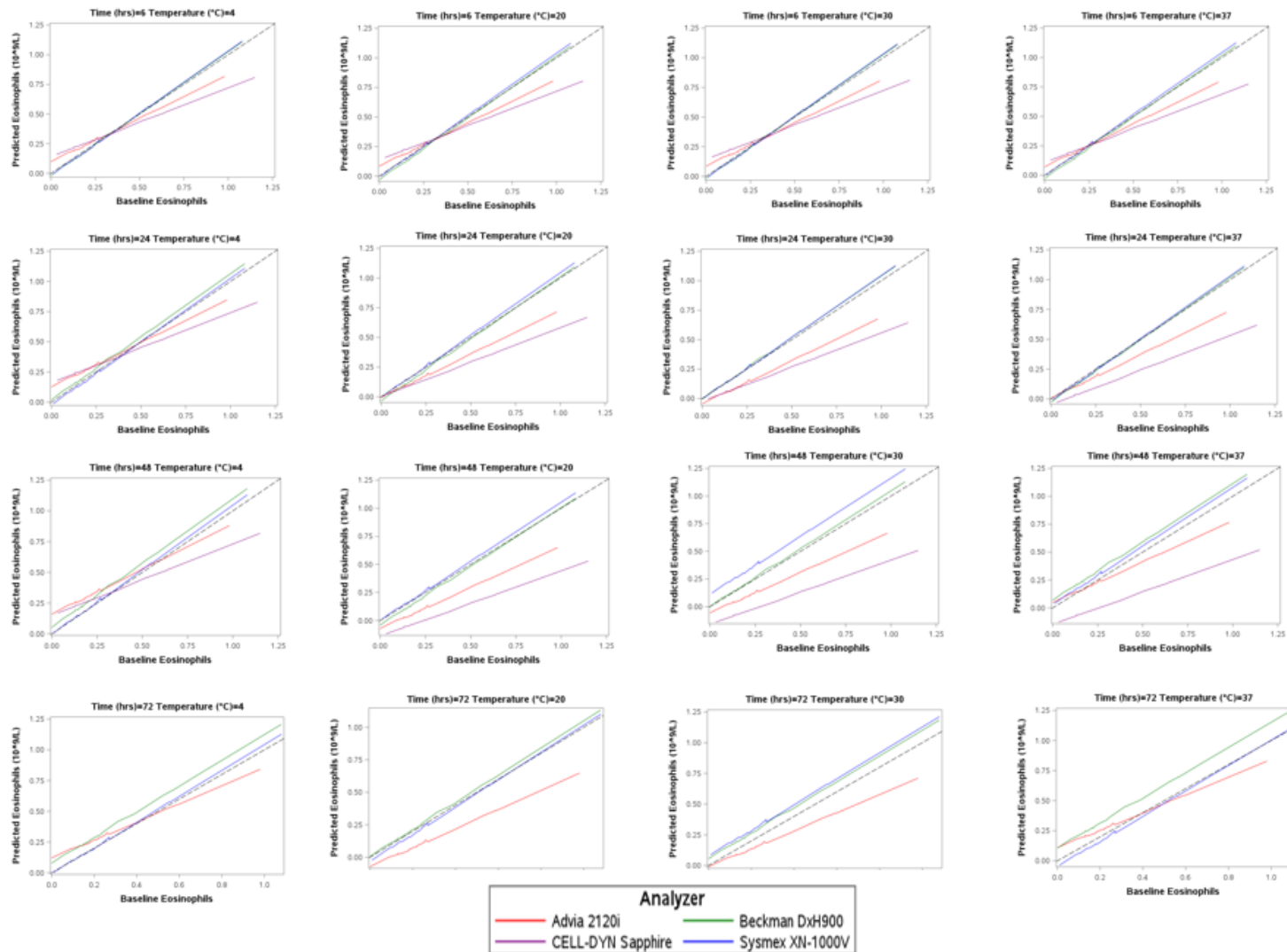

Supplement: S8 Fig — (PDF) [file pone.0301845.s018.pdf]

## ADVIA 2120i scattergrams – Asthmatic donor

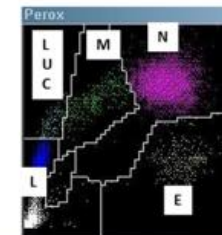

+4°C

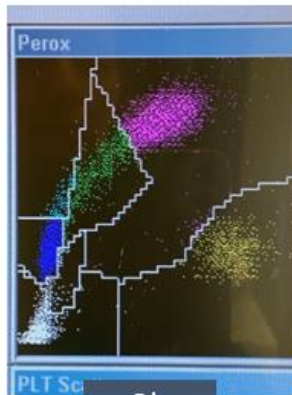

3h

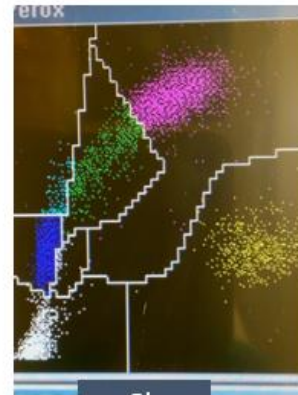

6h

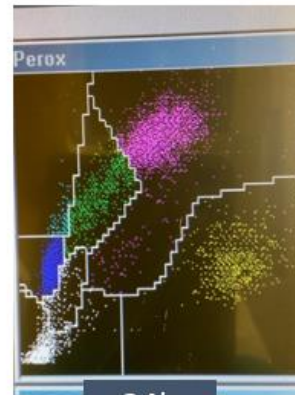

24h

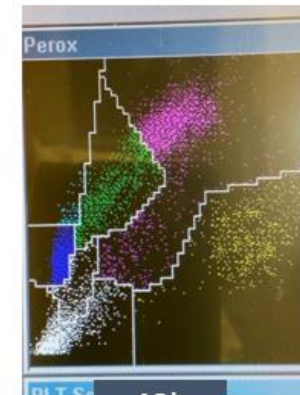

48h

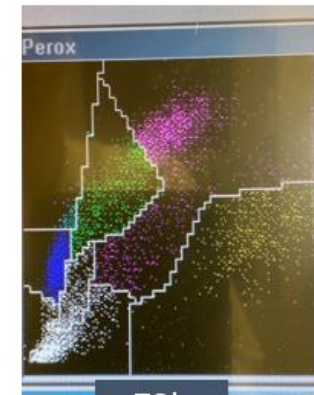

72h

+20°C

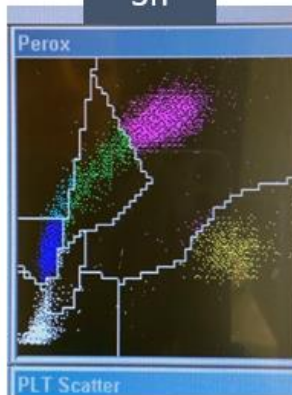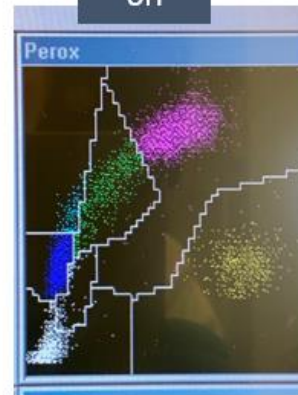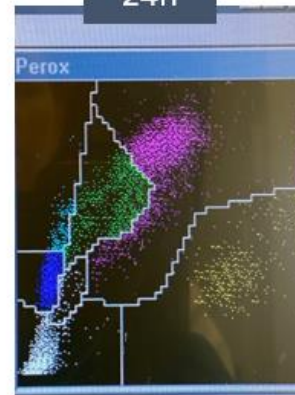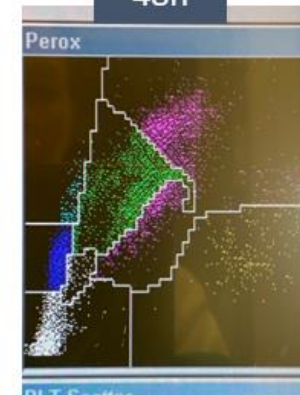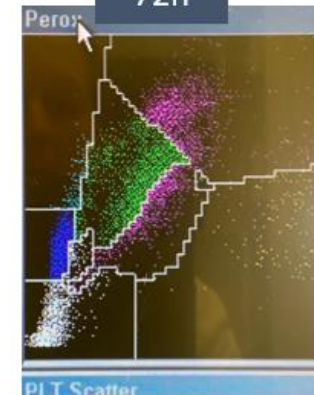

Supplement: S9 Fig — (PDF) [file pone.0301845.s019.pdf]

A

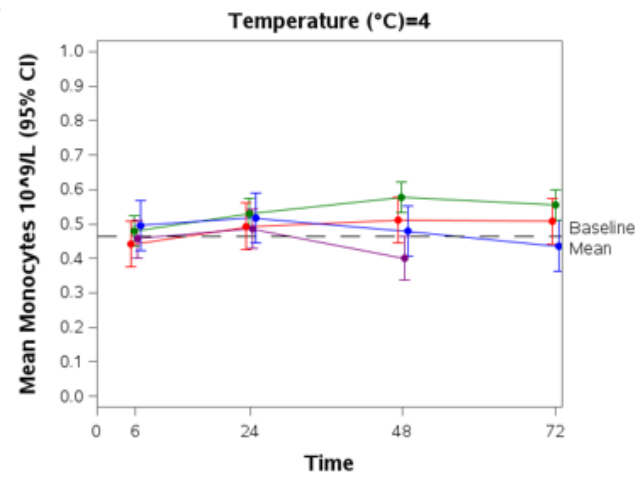

B

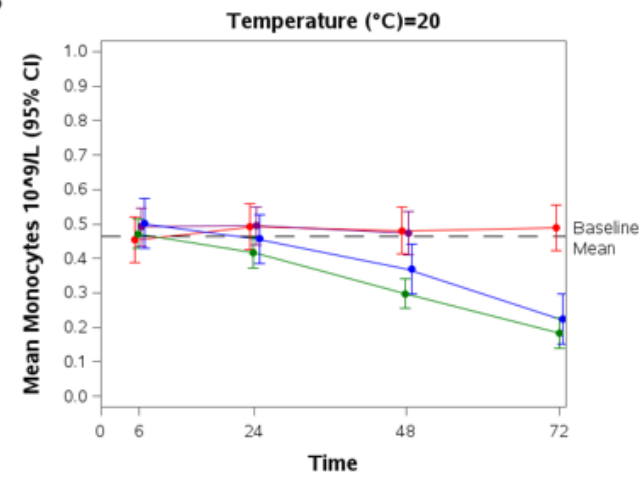

C

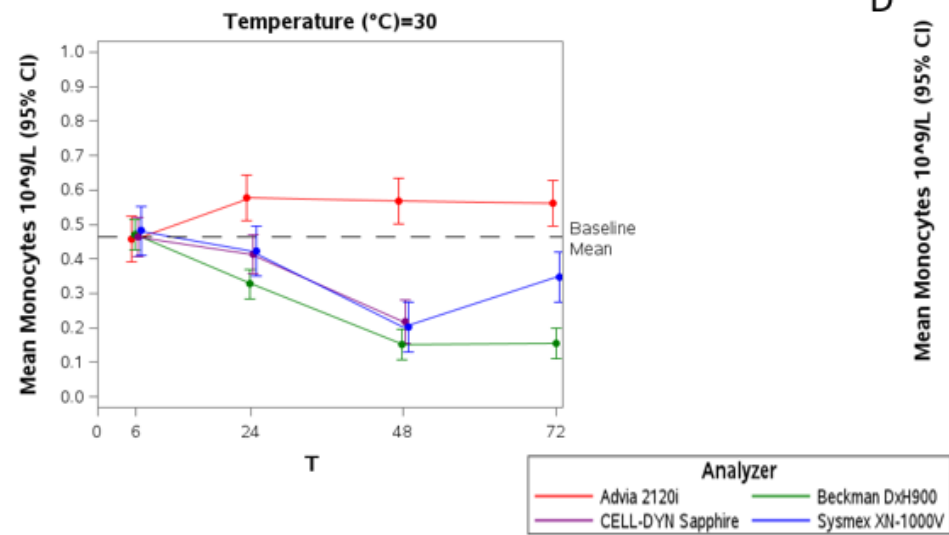

D

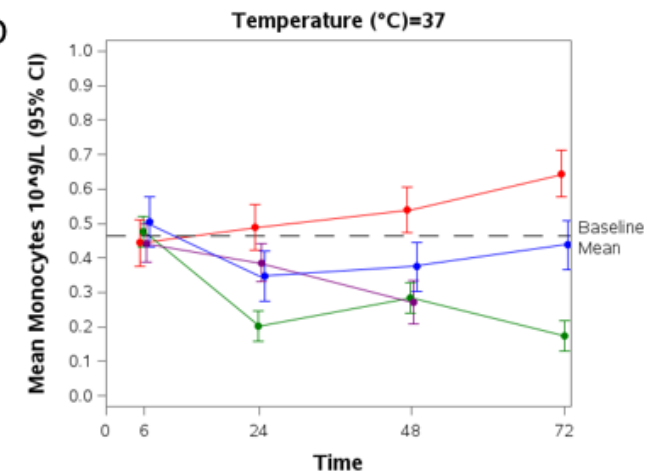

Supplement: S10 Fig — (PDF) [file pone.0301845.s020.pdf]

A

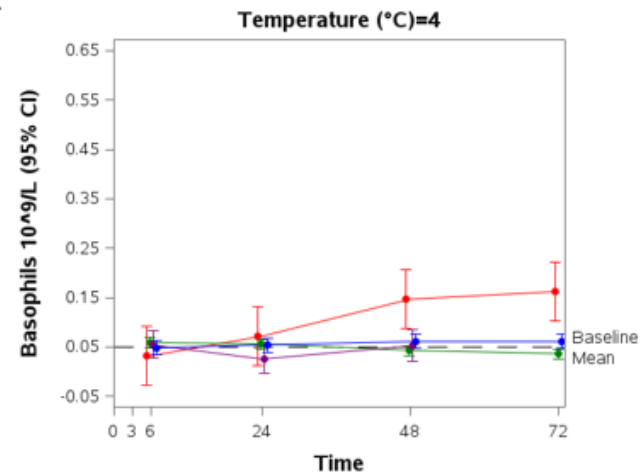

B

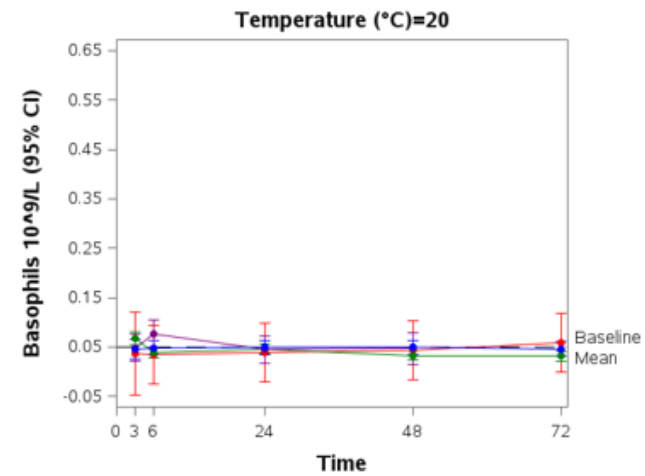

C

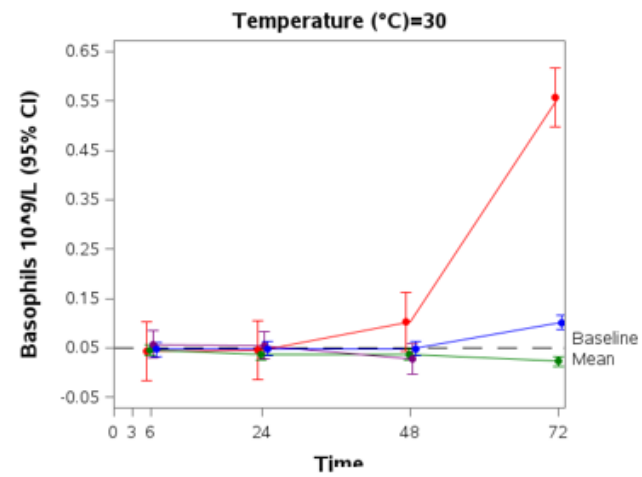

D

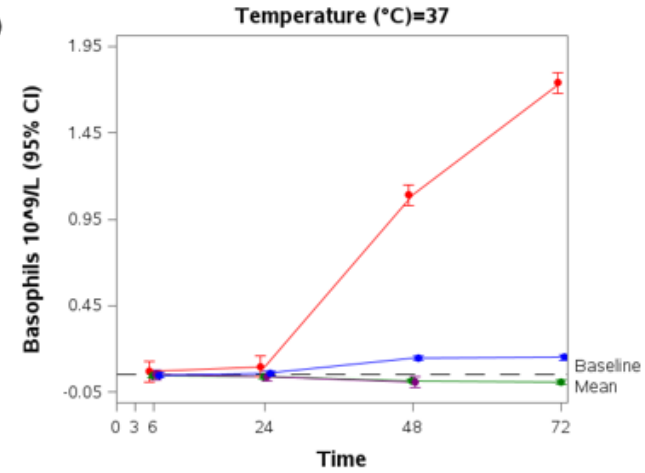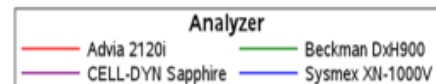

Supplement: S11 Fig — (PDF) [file pone.0301845.s021.pdf]

A

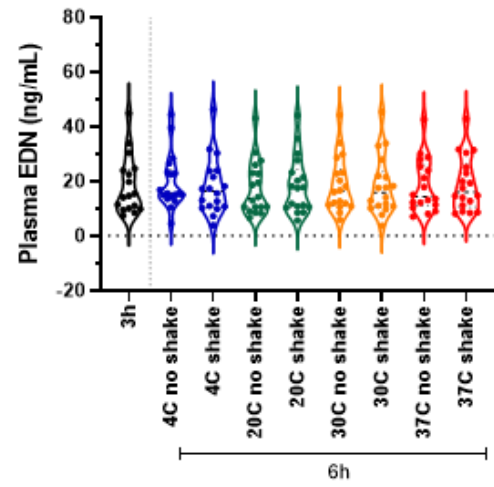

B

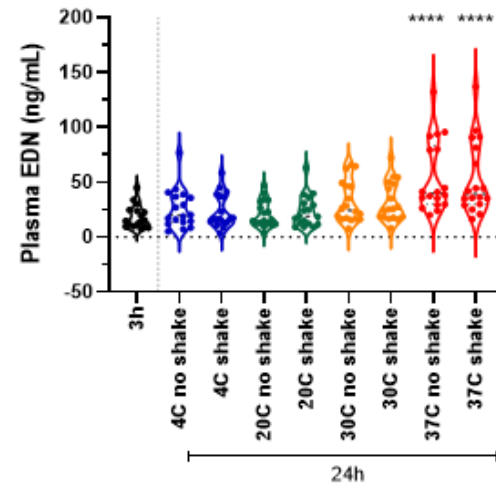

C

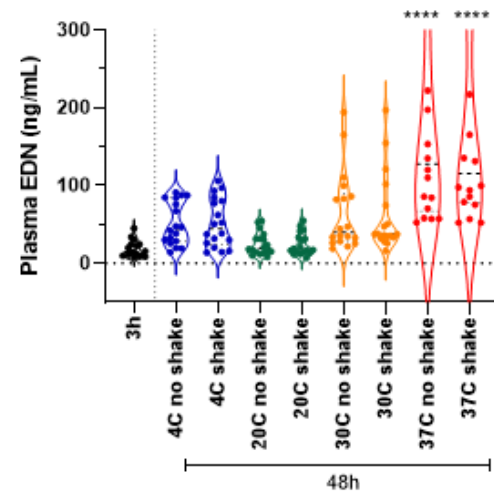

D

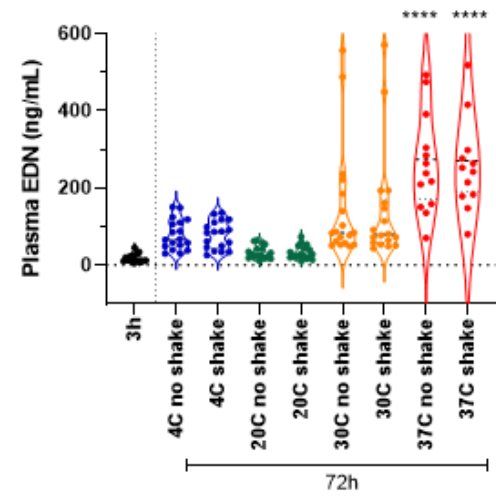

Supplement: S13 Fig — (PDF) [file pone.0301845.s023.pdf]

## A - for eosinophils

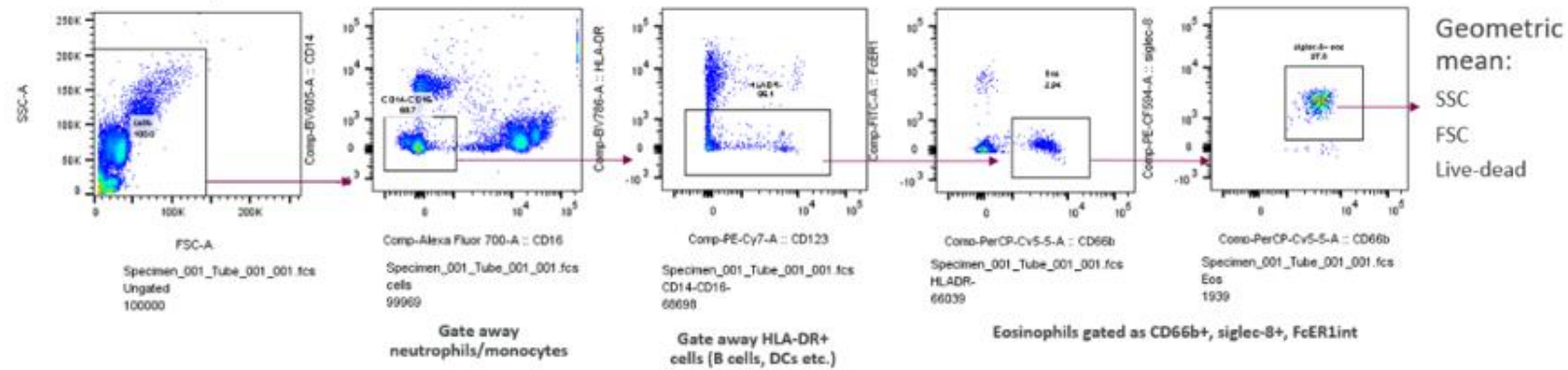

## B - for live eosinophils

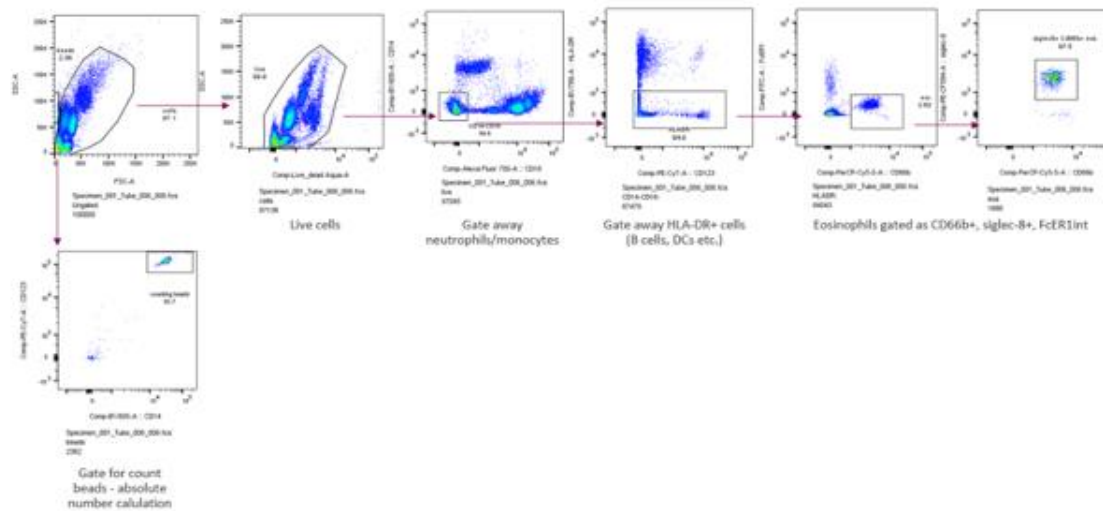

Supplement: S14 Fig — (PDF) [file pone.0301845.s024.pdf]
