# Supplementary material for: A comparative study of blood cell count in four automated hematology analyzers: An evaluation of the impact of preanalytical factors
Source: PLoS One. 2024 May 24;19(5):e0301845. doi: 10.1371/journal.pone.0301845 (PMC11125483; doi:10.1371/journal.pone.0301845)
Supplement: S6 Fig — (PDF) [file pone.0301845.s016.pdf]

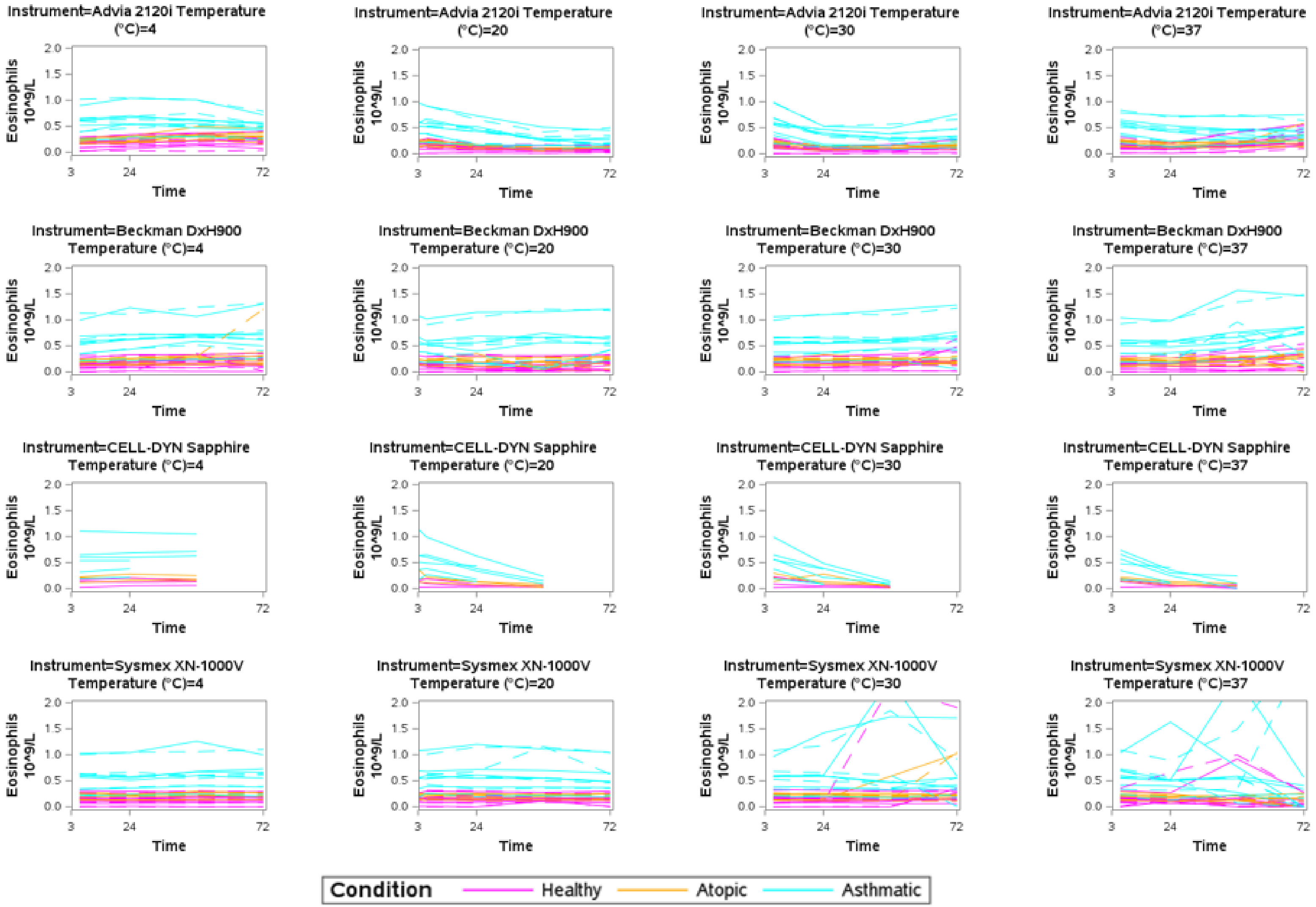
Instrument=Advia 2120i Temperature (°C)=4
Instrument=Advia 2120i Temperature (°C)=20
Instrument=Advia 2120i Temperature (°C)=30
Instrument=Advia 2120i Temperature (°C)=37
Instrument=Beckman DxH900 Temperature (°C)=4
Instrument=Beckman DxH900 Temperature (°C)=20
Instrument=Beckman DxH900 Temperature (°C)=30
Instrument=Beckman DxH900 Temperature (°C)=37
Instrument=CELL-DYN Sapphire Temperature (°C)=4
Instrument=CELL-DYN Sapphire Temperature (°C)=20
Instrument=CELL-DYN Sapphire Temperature (°C)=30
Instrument=CELL-DYN Sapphire Temperature (°C)=37
Instrument=Sysmex XN-1000V Temperature (°C)=4
Instrument=Sysmex XN-1000V Temperature (°C)=20
Instrument=Sysmex XN-1000V Temperature (°C)=30
Instrument=Sysmex XN-1000V Temperature (°C)=37
Eosinophils 10^9/L
0.0
0.5
1.0
1.5
2.0
Time
3
24
72
Condition
Healthy
Atopic
Asthmatic
