# Supplementary material for: A comparative study of blood cell count in four automated hematology analyzers: An evaluation of the impact of preanalytical factors
Source: PLoS One. 2024 May 24;19(5):e0301845. doi: 10.1371/journal.pone.0301845 (PMC11125483; doi:10.1371/journal.pone.0301845)
Supplement: S7 Fig — (PDF) [file pone.0301845.s017.pdf]

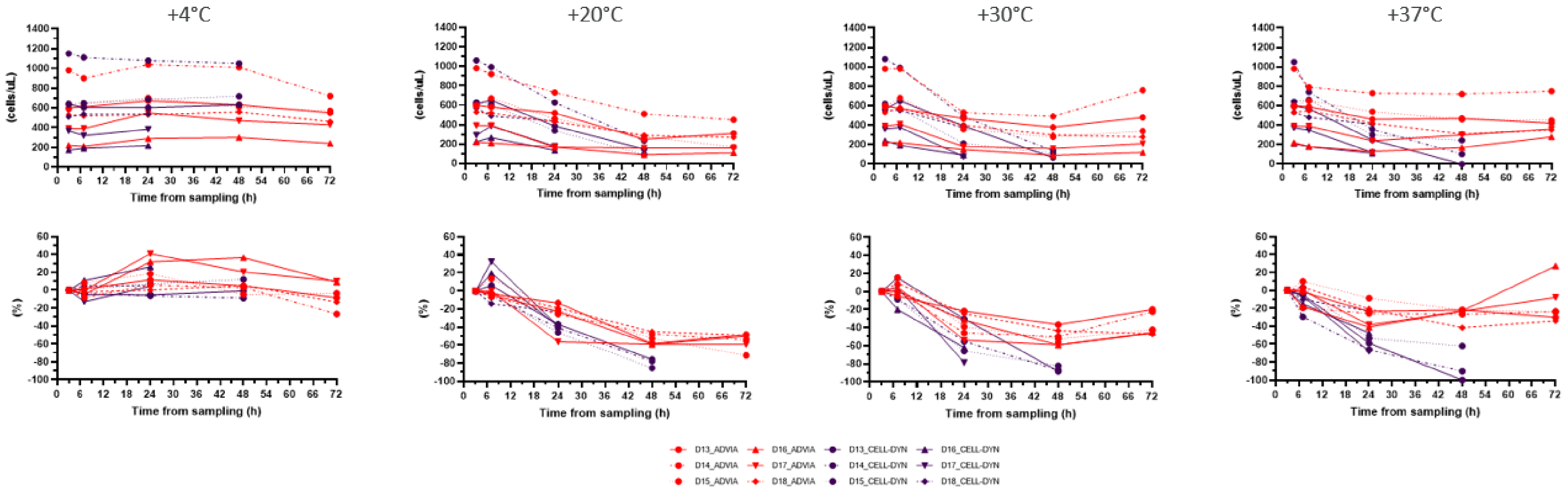
+4°C
+20°C
+30°C
+37°C
(cells/uL)
(%)
Time from sampling (h)
D13_ADVIA
D14_ADVIA
D15_ADVIA
D16_ADVIA
D17_ADVIA
D18_ADVIA
D13_CELL-DYN
D14_CELL-DYN
D15_CELL-DYN
D16_CELL-DYN
D17_CELL-DYN
D18_CELL-DYN
