# Supplementary material for: A comparative study of blood cell count in four automated hematology analyzers: An evaluation of the impact of preanalytical factors
Source: PLoS One. 2024 May 24;19(5):e0301845. doi: 10.1371/journal.pone.0301845 (PMC11125483; doi:10.1371/journal.pone.0301845)
Supplement: S12 Fig — (PDF) [file pone.0301845.s022.pdf]

A

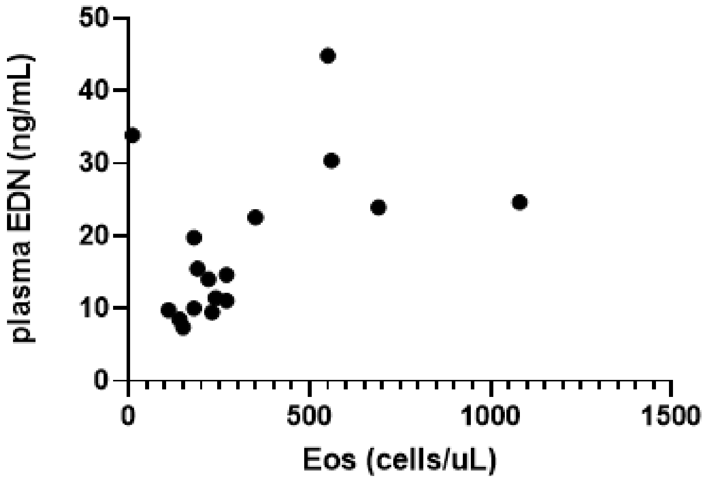

Spearman r	
r	0.5239
95% confidence interval	0.04240 to 0.8079
P value	
P (two-tailed)	0.0327
P value summary	*

B

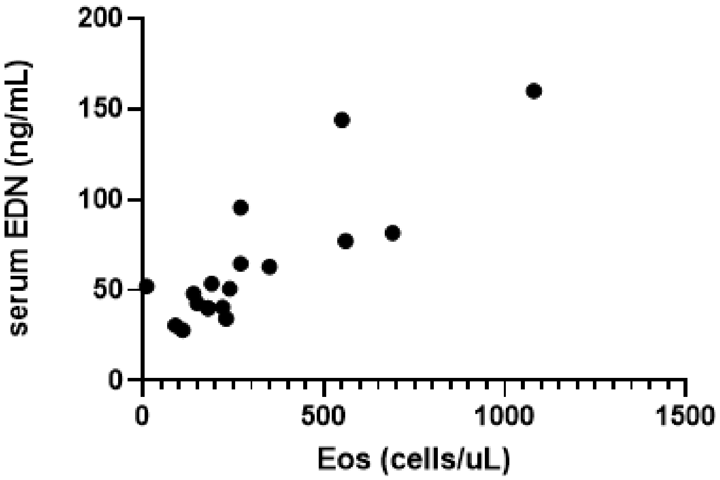

Spearman r	
r	0.7800
95% confidence interval	0.4810 to 0.9164
P value	
P (two-tailed)	0.0001
P value summary	***
