# Supplementary material for: A comparative study of blood cell count in four automated hematology analyzers: An evaluation of the impact of preanalytical factors
Source: PLoS One. 2024 May 24;19(5):e0301845. doi: 10.1371/journal.pone.0301845 (PMC11125483; doi:10.1371/journal.pone.0301845)
Supplement: Attachment — Submitted filename: RESPONSE TO REVIEWERS_Dec 2023.docx [file pone.0301845.s025.docx]

RESPONSE TO REVIEWERS

PONE-D-23-30649

A comparative study of blood cell count in four automated hematology analyzers: an evaluation of the impact of preanalytical factors

Response to the Editor/Journal requirements:

1. We have reformatted the manuscript according to PLOS ONE's style requirements and hope nothing has been missed – if so, please let us know so we can correct

2. Regarding the ‘Funding Information’ and ‘Financial Disclosure’ – this work is conducted by AstraZeneca employers within their working remits and collaborations with Krefting Institute, Sahlgrenska and Beckman Coulter. No grants were received and only payment for the conducted work by Krefting, Sahlgrenska and Beckman Coulter (loan/transportation of the DxH900 platform) was paid by AstraZeneca. We do not see where the information is not matching.

3. Regarding the competing interest, this totally applies: "This does not alter our adherence to PLOS ONE policies on sharing data and materials.” - but we do not see where this information should be added.

4. The previously “data not shown” regarding scattergrams from the ADVIA 2120i platform has been sorted by adding a supplementary figure (S9 Fig) to demonstrate the infiltration of neutrophils into the eosinophil gate.

5. All of the 14 included references are found upon searching the internet and also by means of PubMed and included in the manuscript via EndNote X9. Hernandez et al 2020 from the M&M section was unfortunately missing in the reference list previously but is now inserted in the revised version. We are also referring to the user manuals for the machines in the supplementary material – these are not publicly available as published material but can be found via the home page of the specific provider. For the benefit of the reader, we have extracted to most important information regarding their respective technology, however specific details incl. algorithms are business confidential information and not shared by the providers.

Response to the Reviewers:

Reviewer #1: The authors compare 4 hematology Analyzers in whole cell blood count, but most of clinical laboratory has one or two analyzer that they are working.
The authors neither experiment nor discuss about RBC, HCt, Hgb and RBC index's. may be they will do it in their next manuscript.
the aim of this study is not clear.

Response: We focused our study on the White Blood Cells as stated in the beginning of the paper, and albeit more information is achieved in the analysis for all hematology platforms, we did not summarize the information for RBCs, HCt, Hgb etc. This could be done in a separate project by using the existing data.

We have clarified (Abstract and Introduction) 1) the gap in knowledge regarding aging blood samples and pre-analytical factors in the public domain and 2) the aim of our study to provide solid data that would help with future cell differential evaluations.

Reviewer #2: Well written and well formatted article. Technically sound.Excellent work.
The methodology is sound,However the sample size could have been higher to establish more conclusive findings.This helps to identify possible pre analytical variables and their influence on outcomes

Response: We agree, it would have been preferred to have a larger number of donors in our study, but for technical limitations (the ability to analyse samples within a certain time frame), we had to limit the study to n=6 per experiment. We had previous knowledge internally regarding variability in the measurements and also whether there seemed to be differences between high/low eos subjects, in disease and in healthy individuals. Our power calculations, taking into account the repeated measures per sample and the multiple conditions, indicated that an n=18 would be adequate to compare between platforms for solid results and the n=6 per subject condition would be enough to identify any major changes similar to the size of differences found between platforms. As seen in the results (initial statistical evaluation), the subject condition does not affect the ability of the platforms to identify and count white blood cell differentials, nor did the mechanical stress make any difference, so these results have been averaged. Taken together, each donor has very strong data and this is included and explained in the analysis section.

We have extended the section on ‘Limitations of the study’ to highlight that with the identification of no interference of disease status or effect of mechanical damage, the sample size was extensively increased by merging results for the different conditions between platforms.
