# Supplementary material for: A comparative study of blood cell count in four automated hematology analyzers: An evaluation of the impact of preanalytical factors
Source: PLoS One. 2024 May 24;19(5):e0301845. doi: 10.1371/journal.pone.0301845 (PMC11125483; doi:10.1371/journal.pone.0301845)
Supplement: Attachment — Submitted filename: RESPONSE TO REVIEWERS_240313.docx [file pone.0301845.s026.docx]

RESPONSE TO REVIEWERS

PONE-D-23-30649

A comparative study of blood cell count in four automated hematology analyzers: an evaluation of the impact of preanalytical factors

Response to the Reviewer #3:

1- **The authors need to justify the focus on eosinophils. Perhaps stating in the introduction.**

Thanks for highlighting this – we have made changes in both the Abstract and Introduction to justify the focus on eos:

***Abstract***, line 37-38: “…using four of the most utilized commercial platforms, focusing on the assessment of eosinophils given its importance in asthma management.” This helps to understand the study design in the upcoming Method section (patients with eosinophilic asthma).

***Introduction***, line 68: “…, mostly issues for basophils, eosinophils and neutrophils, which was later confirmed by…” added in order to set the scene for our interest.

Furthermore, in the ***Introduction*** p.6, line 86-87, we have added: “An accurate eosinophil count is for example important in treatment selection of asthma patients.”

***Discussion***, expansion on the rationale for the focus on eosinophils: “Focusing on the eosinophils, given its importance in asthma management, there are significant differences in the method….”

**2- The experimental design is a little confusing and hard to follow. I suggest adding a flow chart of the different participants and their characteristics and different experiments and manipulations done.**

Thanks for a good suggestion, we have prepared a schematic of the schedule of sampling and assessments which is now included in the manuscript as Schedule of Assessments in the M&M section.

M&M page 6, line 103-104: “Demographics for the 18 participants in the study are shown in S1 Table and in a schematic of the Schedule of Assessments.”

**3- Please define what you mean by baseline in the methods section.**

Thanks – we have clarified by insertion of the times for the different assessments also in the method section:

Page 7, line 106-109: “…, #367838 BD Vacutainer) for cell differentials (CBC) at 5 time points: 3h (baseline), 6h, 24h, 48h & 72h, flow cytometry analysis (at 3, 24, 48 & 72h) and plasma collection (all time points), and in one 3.5 mL serum separation tube (Serum separator tubes, SST™ II advance, BD Vacutainer® ref 368498 (Becton Dickinson Sweden AB)) for serum collection (baseline (3h) only).”

**4- In line 109 it is mentioned that two tubes from each donor were put in 4 different temperature. Were each tube split into two different tubes in order to have them in 4 different temperatures? This is not clear. There are many instances where the methodology of handling the samples is not clear. Therefore, it will be hard for other scientists to reproduce the protocol used.**

With the schematic of the schedule of sampling and assessments, plus the clarification for timepoints for the different tubes and assessments in the method section, we hope this is now easier to follow.

**5- In line 122, RT, which I assume is refereeing to room temperature, was not defined prior to this point**

Thanks – RT was explained for when room temperature is mentioned the first time in the M&M section:

Page 7, line 116: “… (at 4°C), in a box on the bench (room temperature (RT) of 20°C), and…”

**6- I have a concern regarding patch effects of the different experiments run at different places and different times and yet they were compared to each other in the results. Is there a way statistically to control for that?**

It is a limitation of the study that we were unable to install all four platforms in the same lab but instead needed to collaborate with the nearby hospital Clinical Chemistry department, only 5 kilometres away (brought up in the Discussion section). The hospital is on the other hand across the street from the Krefting Center from where the blood from the eosinophilic asthmatics were sampled, so blood has been carefully transported either from AZ (healthy donors) to the Hospital University area or the other way around within the time frame of the 3h from sampling to first analysis. All samples have been run **at the same time** in the different analyzers, but by different well-trained technicians. We cannot control for that samples were run at different places statistically; thus results will be **confounded with the analyzer, in this case the CELL-DYN Sapphire placed at the Sahlgrenska university hospital**. We believe this is however a minor issue as our findings are consistent with what was found previously by a Norwegian hospital lab using the SYSMEX, CELL-DYN and ADVIA platforms and 2 temperatures only (personal communication and knowledge sharing, brought up in the Acknowledgement section). Furthermore, the CELL-DYN Sapphire analyzer was added to the study design as a positive control, knowing that it would greatly underestimate the cell counts for when post-sampling conditions were not optimized for the platform of choice. For practical reasons, we further had to settle with fewer donors and timepoints in this platform, which is brought up as a limitation in both the M&M, Result and Discussion sections.

**7- In Line 133, “(w/wo)” means with or without atopy? Is there a reason for not using one, either with or without, or using both groups separately; so the healthy will have n=6 (3+3).**

Yes, w/wo means ‘with or without’ and we have now spelled it out in this parenthesis. The reason for having both of these healthy groups is the fact the healthy group *without* atopy is likely to have no to very low T2 inflammation and eosinophil counts, whereas the atopic healthy group (the ‘*with’* group) is more likely to have slightly higher eos counts and potentially activated cells – we designed the study to have n=6 in both these groups which, if there were no differences, would make n=12 for the healthy group – and this is indeed what we found. Likewise, if the eosinophilic asthmatics were also not different to the healthy groups with respect to the activation of eosinophils, we would have n=18 in the study (which was also found to be the case – there was no impact of disease status on the way the hematology analyzers managed to count the white blood cells including eosinophils). The n=3+3 was run at 2 separate experiments to better randomize the groups over the experimental days, we still have n=6 per group, healthy non-atopic, healthy with atopy, and eosinophilic asthma, in total n=18.

**8- For the flow cytometry and EDN assay, were there technical replicates? If not please justify.**

We did not run technical replicates in the FACS as we were limited to the amount of samples that could be run in one day for this technology platform (having a lower throughput than the hematology analyzers). We chose to analyze all donors and groups at all timepoints except for the 6h timepoint, and also restricted the temperature groups to 4, 20 and 30°C as we knew from a pilot test that eos were highly affected at 37°C, to best capture the range where eosinophils started to get activated and/or induce cell death. We did also not include technical replicates for the Flow Cytometry samples as in our experience, the biological variability between individual donors is much greater than any variation introduced by sample handling. Samples were stained in plates using the same antibody cocktail mixture, thus all samples were treated in an identical manner before acquisition. Moreover, the daily performance of the instrument was carefully monitored by using Cytometer Setup and Tracking (CS&T) beads to ensure sure consistent and reproducible results with minimal variability. Moreover, we further deselected the test tubes exposed to mechanical stress based upon the limitation of possible sample numbers per day.

With regards to EDN, we analyzed all plasma samples at all timepoints and conditions (making 594 samples) plus the 18 serums at baseline, but only in singlicates. We see good consistency and pattern within donors, and duplicates in the standard curve, indicating low variability in assay performance. We also did not perform any statistical evaluation for the EDN results except for intra-analysis correlations and trends in increased release of EDN with expression of activation markers from the FACS.

**9- In line 167, it is indicated that the diluted samples had an upper range of 640 ng/ml which is outside the standard curve. It is not very clear this section in the methods as there are many different brackets and they are not always properly used and effects the understanding the sentences. This is seen all-over the manuscript and needs revisions.**

The samples were diluted 20-fold with the standard curve having a max of 32ng/mL (ie 640ng/mL when corrected for by the dilution factor) – all samples and standard curve measures were thus within the generated standard curve. We have modified the sentence in the manuscript to avoid confusion and it now reads: “100 μL of the calibration curve standards (0.25–32 ng/mL), controls (C1:[0.7 ng/mL] and C2:[2.94 ng/mL]) and samples diluted 20-fold in assay buffer (#6811, Immundiagnostik AG) were added to the plate wells. Standards were run in duplicates whereas samples and controls were run in singlicates.”

Thanks for constructive feedback, we have corrected all parentheses where mismatching and tried to make sure they are properly used throughout the result section. For the EDN result section, it now reads: “Eosinophil-derived neurotoxin (EDN), a commonly used biomarker of eosinophil activation, was relatively stable in samples kept at 20°C up to 72h post sampling (Fig 7, S10 Table), but found to be increased in plasma from 48 hours post-sampling for samples kept at higher temperatures. The difference between mean at baseline (3h, 20°C) and 48h at 37°C was -224ng/mL (95%CI -322, -125ng/mL). By 72 hours at 30°C it was -132ng/mL (-231, -34ng/mL) and at 37°C, -478ng/mL (-579, -376ng/mL).”

**10- The results section is disorganized and very hard to follow. it needs to be organized with clear subheadings and better flow. For example, in the first section of the results, after the demographics of the participants, is not very clear what experiments our question is being answered especially that it feels like some the results are just repeated in other sections. Moreover, the description of the results between the different platforms and the different temperature and times need to be written in a more clear. For example, when the increase is mentioned by X (y-z) are you referring to a percent increase and a range. This need to be added in the statistical analysis section, where you mention that data are presented as ………. The results section needs major reorganization to help the readers follow the logic of the experiments.**

Thanks for constructive feedback – We have carefully gone through the Result section and made simplifications (see tracked changes in the manuscript). There are no repeats in the different sections as we structured the results based upon the best way (we felt) in how to present the immense data in Figures and Tables/Supplementary materials taking it step by step from baseline to the different temperatures evaluated, followed by the different conditions and biomarkers of activation. Based upon your suggestion, we have added more subheadings to better see what the upcoming section is about and believe this enhances the understanding of the findings. Each descriptive section of cell count starts by giving the explanation for the values, see “difference in LS means in cells x10^9^/L (95% confidence interval)”, not to have to repeat it with each statement and save space. All other places have their respective unit given adjacent to its numbers presented.

**11- The discussion need to describe the results in more details. For example, in line 358, the authors need to rewrite this to better descibe their findings in the different experiments. Also, in line 366, the authors state that leukocytes are sensitive to external temperatures; was this based on the their data or from the literature? They need to specify that.**

Thanks for additional constructive feedback, we have made multiple clarifications in the Discussion section and further included the Unelli et al paper as reference for the sensitivity of leukocytes to *ex vivo* temperatures. Please refer to the tracked changes in our resubmitted manuscript.

**12- Discuss the significance of examining eosinophils specifically.**

Thanks. we have made a few additions to the paper to highlight the eosinophils (see our response to comment 1) – we do analyse and discuss all cell differentials so would like to keep the heading and message of the paper on all white blood cells, however, the study design clearly shows the main focus on eosinophils. We have thus increased the significance and justification for optimal eosinophil assessment in both the abstract, introduction and discussion.
